# Effect of an oat cookie enriched with bean flour biotransformed by *Pleurotus ostreatus* on nutritional and inflammatory status in male CD-1 mice

**DOI:** 10.3389/fnut.2026.1824912

**Published:** 2026-08-27

**Authors:** Xiomara Cardona, Heriberto Castro, Paulina Álvarez Guzmán, Juan José Acevedo Fernández, Edith Espinosa Páez

**Affiliations:** 1 Universidad Autónoma de Nuevo León, Facultad de Salud Pública y Nutrición, Centro de Investigación en Salud Pública y Nutrición, Laboratorio de Genética y Biología Molecular, Monterrey, Mexico; 2 Universidad de Monterrey, Escuela de Ciencias Aliadas de la Salud, Departamento de Nutrición, Monterrey, Mexico; 3 Universidad Autónoma del Estado de Morelos, Facultad de Medicina, Laboratorio de Electrofisiología y Bioevaluación Farmacológica, Cuernavaca, Mexico

**Keywords:** biotransformation, functional foods, hypercaloric diet, low-grade inflammation, metabolic alteration

## Abstract

**Introduction:**

Metabolic alterations associated with obesogenic diets are closely linked to chronic low-grade inflammation, highlighting the need for functional foods that can complement conventional dietary strategies. This study evaluated whether an oat cookie enriched with bean flour biotransformed by *Pleurotus ostreatus* modulated metabolic, tissue, and inflammatory parameters in male CD-1 mice under standard, hypercaloric, and dietary-reversal conditions.

**Methods:**

Forty-five male CD-1 mice were allocated to nine groups (*n* = 5 per group) according to a metabolic alteration phase and a supplementation phase. During Phase 1, mice received either a standard diet (STD) or a hypercaloric diet (HYP) for 12 weeks without cookie supplementation. During Phase 2, mice either continued their original diet or transitioned from the hypercaloric to the standard diet (HYPSTD) and received a biotransformed cookie (B), a compositionally comparable non-biotransformed cookie (NB), or no cookie (CF) for 8 weeks.

**Results:**

During the first phase, no significant differences were detected in body weight, fasting glucose, triglycerides, or glucose tolerance, although HYP mice consumed more lipids and energy. During the supplementation phase, STD B mice exhibited lower liver- and adipose-tissue-to-body-weight ratios than STD CF mice (*p* < 0.05). In HYP mice, both cookie-supplemented groups showed lower serum interleukin-6 concentrations than the CF group (*p* < 0.05). Within the HYPSTD condition, B mice maintained a lower body weight than CF mice from weeks 12–20 (*p* < 0.05), while IL-6 concentrations were lower in B than in NB mice (*p* < 0.005). Glucose concentration at 15 min during the OGTT was positively correlated with final body weight, and IL-6 was inversely correlated with IL-10.

**Discussion:**

Unlike previous studies focused primarily on ingredient characterization or isolated bioactive compounds, this study evaluated the effects of an oat cookie enriched with *P. ostreatus*-biotransformed bean flour on metabolic, tissue, and inflammatory outcomes in male CD-1 mice under standard, hypercaloric, and dietary-reversal conditions. These findings suggest that *P. ostreatus*-biotransformed bean flour has potential as a functional ingredient for bakery products and may modulate selected tissue and inflammatory outcomes.

## 1 Introduction

Obesity and type 2 diabetes mellitus are among the leading cardiometabolic diseases worldwide and are characterized by a state of chronic, low-grade inflammation associated with alterations in inflammatory biomarkers, such as tumor necrosis factor-alpha (TNF-*α*) and interleukin-6 (IL-6) (1, 2). Diet is a major modifiable factor influencing their development and progression, and there is increasing interest in dietary strategies capable of modulating inflammation and improving metabolic health (2). Accordingly, functional foods have emerged as promising nutritional approaches because they provide bioactive compounds with antioxidant and anti-inflammatory properties that may contribute to the prevention and management of metabolic disorders (3).

Among these strategies, the development of functional foods enriched with plant-based ingredients has gained considerable attention as a means of improving the nutritional quality of commonly consumed products. Composite flours based on whole grains and legumes have emerged as promising ingredients for enhancing the content of dietary fiber, proteins, and health-promoting compounds while maintaining suitable technological and sensory characteristics in bakery products (4–6). Oats and beans are especially attractive because of their complementary nutritional composition, providing *β*-glucans, avenanthramides, phenolic acids, anthocyanins, and bioactive peptides associated with antioxidant, anti-inflammatory, and metabolic health benefits (3). Consequently, innovative technological approaches have been explored to further enhance the bioavailability and biological potential of these compounds.

Among the approaches used to improve the value of plant-based ingredients, microbial biotransformation has emerged as a promising alternative for increasing the nutritional quality and bioavailability of bioactive metabolites. In this context, *Pleurotus ostreatus* (oyster mushroom) stands out because of its rapid growth, ability to colonize lignocellulosic substrates, and capacity to produce extracellular enzymes that facilitate hydrolysis of the food matrix and the release of bioactive compounds (7, 8). This process increases the availability of metabolites such as *β*-glucans and ergothioneine, improves protein digestibility, and reduces antinutritional compounds while preserving these benefits after thermal processing (9, 10). Previous work by our research group demonstrated that fermentation with *P. ostreatus* increased antioxidant capacity, total phenolic content, and protein digestibility in *Phaseolus vulgaris* and *Avena sativa flours* (11). Furthermore, cookies produced with these biotransformed ingredients retained functional properties after baking and simulated gastrointestinal digestion (12).

Nevertheless, developing enriched functional foods presents important challenges. The addition of plant powders or alternative flours can modify dough rheology, texture, color, flavor, water and oil retention, storage stability, and consumer acceptability. These ingredients may also provide useful technological and biological functions when incorporated at appropriate concentrations. For example, Ünal Turhan et al. found that adding carob powder to probiotic peanut butter improved oil retention, reduced oil loss, and supported the viability of *Lacticaseibacillus rhamnosus*, while the products maintained acceptable sensory characteristics during storage (13). Processing conditions, particularly heating and baking, may also affect the stability, accessibility, and activity of bioactive compounds. Furthermore, improvements in chemical composition, technological performance, or *in vitro* properties do not necessarily demonstrate that the finished product will produce physiological effects after consumption. Consequently, functional-food research should extend beyond formulation and sensory characterization to include assessments of bioaccessibility, biological activity, and *in vivo* metabolic responses.

Despite advances in the development of biotransformed flours and evidence demonstrating improvements in their nutritional composition and technological properties, limited information is available on whether these advantages are retained after incorporation into baked products and subsequently translate into *in vivo* biological effects under conditions of diet-induced obesity. Previous studies have focused primarily on the physicochemical and nutritional characterization of biotransformed flours, whereas considerably less attention has been given to evaluating their metabolic and inflammatory effects following incorporation into baked foods. Demonstrating that the benefits achieved through biotransformation remain biologically active after processing and consumption is a crucial step toward the development of functional foods with potential applications in the prevention and management of metabolic diseases. In this context, the present study evaluated the effects of consuming an oat cookie enriched with *P. ostreatus* biotransformed bean flour on metabolic and inflammatory parameters in male CD1 mice with diet-induced obesity. It was hypothesized that this intervention would promote the recovery of metabolic status and reduce the inflammatory response, particularly when combined with a switch to a standard diet.

## 2 Materials and methods

### 2.1 Animal and experimental design

Three-week-old male CD-1 mice with an average body weight between 17 and 18 grams were obtained from the animal facility of the Universidad Autónoma del Estado de Morelos (UAEM). Animals were housed in polypropylene cages under controlled environmental conditions (21–22 °C, 12-h light/dark cycle), with *ad libitum* access to food and water. Mice were randomly assigned to nine experimental groups (*n* = 5 per group) (14), as follows: standard diet + biotransformed cookie (STD B), standard diet + non-biotransformed cookie (STD NB), standard diet + no cookie (STD CF), hypercaloric diet + biotransformed cookie (HYP B), hypercaloric diet + non-biotransformed cookie (HYP NB), hypercaloric diet + no cookie (HYP CF), hypercaloric diet followed by standard diet + biotransformed cookie (HYPSTD B), hypercaloric diet followed by standard diet + non-biotransformed cookie (HYPSTD NB), hypercaloric diet followed by standard diet + no cookie (HYPSTD CF).

The study comprised two phases. In Phase 1 (12 weeks), mice were exposed to a hypercaloric diet to induce metabolic alterations in hypercaloric groups, while the remaining groups were maintained on a standard diet. No cookie supplementation was provided during this phase. Each cage received 50 g of base diet daily. During Phase 2 (8 weeks), the B and NB groups were offered the corresponding base diet and assigned cookie separately, with each component representing 50% of the total food initially provided. The CF groups received only their corresponding base diet.

During both phases, leftover food was weighed every 24 h using an internally calibrated sensor analytical balance (LB-42AWH; Labotronics Scientific, Wilmington, DE, USA; capacity: 4000 g; resolution: 0.01 g), and average daily intake per mouse was estimated by dividing cage-level intake by the number of animals in the cage (g/day). Food intake data were averaged to determine daily intake (g/day) per mouse across both phases. During the supplementation phase, intake of both cookie and base diet was recorded separately (Table 1). Food spillage was collected and included in the calculation of the remaining food. Results were analyzed and presented every 4 weeks (Phase 1: weeks 1, 4, 8, 12; Phase 2: weeks 12, 16, 20).

**Table 1:** Comparison of diet and cookie consumption during Phase 2 across supplemented groups.

Group	Week 12			Week 16			Week 20		
	Diet	Cookie	*p*	Diet	Cookie	*p*	Diet	Cookie	*p*
STD NB	5.20	5.57	0.885	3.06	3.85	0.24	2.13	3.22	**0.02**
STD B	5.31	5.72	0.915	1.67	2.45	0.25	1.78	2.95	**0.01**
HYP NB	5.17	5.26	0.776	1.70	3.16	**<0.001**	1.79	2.90	**0.02**
HYP B	5.29	6.00	0.13	1.58	2.88	**<0.004**	2.23	2.36	0.83
HYPSTD NB	3.89	5.42	0.318	1.07	3.14	**<0.001**	2.29	3.21	0.11
HYPSTD B	3.80	5.50	0.145	1.38	2.81	**<0.001**	2.74	3.16	0.88

Data are presented by week and supplementation groups. Values are expressed as mean ± standard error of the mean (SEM). Intergroup differences were analyzed using one-way ANOVA followed by Tukey’s *post hoc* test. *n* = 5 per group. STD NB, standard non-biotransformed cookie; STD B, standard biotranformed cookie; HYP NB, hypercaloric non-biotransformed cookie; HYP B, hypercaloric biotranformed cookie; HYPSTD NB, hypercaloric-standard non-biotransformed cookie; HYPSTD B, hypercaloric-standard biotranformed cookie. Bold values meaning is *p* < 0.05.

**Figure 1:**
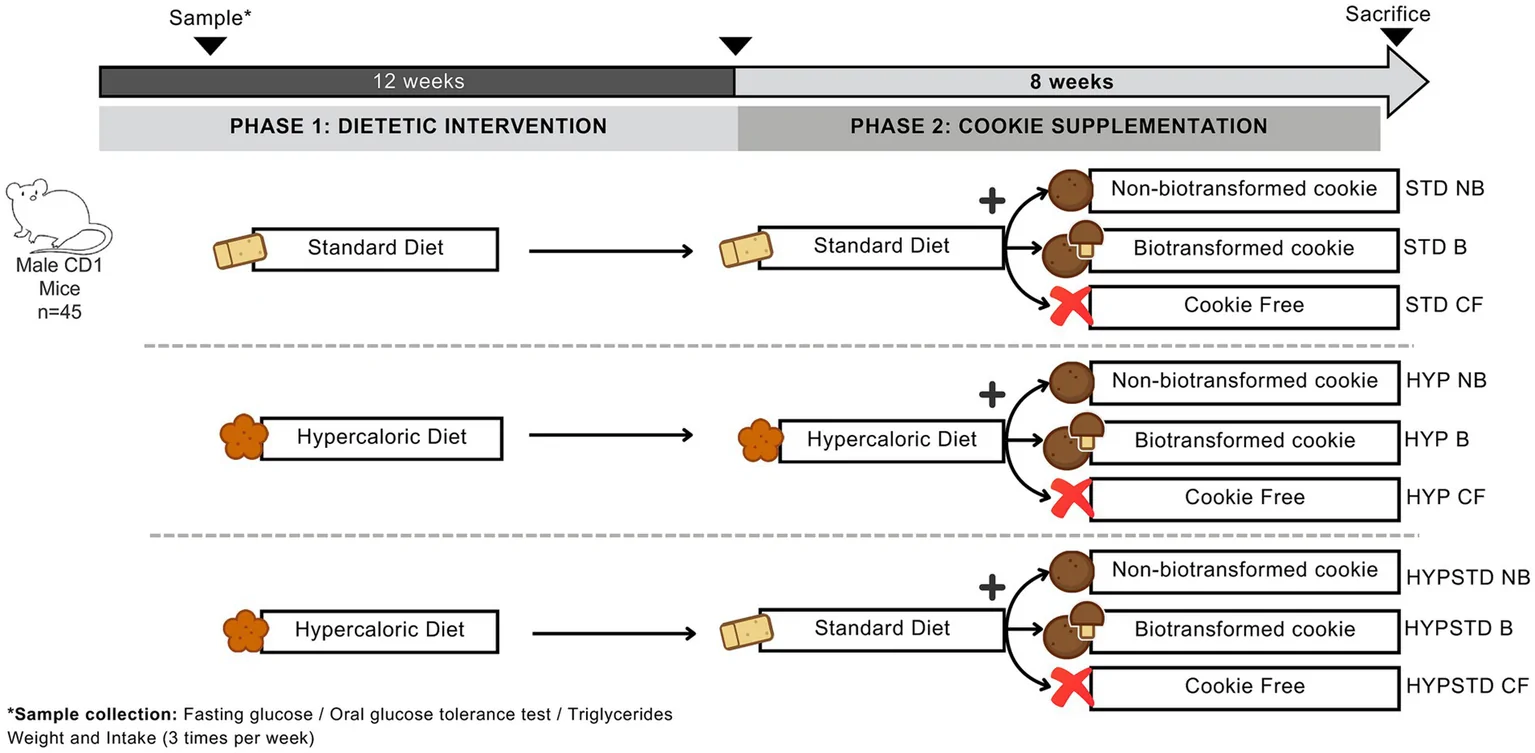
Experimental design. Male CD-1 mice (*n* = 45) were subjected to a 12-week dietary intervention (Phase 1) followed by an 8-week cookie supplementation phase (Phase 2). Animals were assigned to standard (STD), hypercaloric (HYP), or hypercaloric-to-standard diet (HYPSTD) groups. During Phase 2, mice received either a biotransformed cookie (B), non-biotransformed cookie (NB), or no cookie (CF). The base diet and assigned cookie were offered separately, each representing 50% of the total food initially provided. Sample collection included fasting glucose, oral glucose tolerance test (OGTT), triglycerides, and body weight monitoring.

Body weight was measured three times per week using an internally calibrated sensor analytical balance (LB-42AWH; Labotronics Scientific, Wilmington, DE, USA; resolution: 0.01 g) (Figure 1).

All procedures followed the Guide for the Care and Use of Laboratory Animals, eighth edition (15) and the Mexican standard NOM-062-ZOO-1999. The experimental protocol was approved by the Animal Care Committee of the Facultad de Salud Pública y Nutrición (Approval No. 24-FaSPyN-SA-16.TP) and conducted in accordance with the Institutional Biosafety Committee of the Universidad Autónoma de Nuevo León.

### 2.2 Diets

During the metabolic-alteration phase, two types of diets were administered: a standard chow diet (LabDiet, St. Louis, MO, USA) and a hypercaloric diet prepared by combining 100 g of ground Rodent Diet 5,001 with 50 g of commercial unsalted cow’s-milk butter (Gloria; Cremería Americana, S.A. de C.V., Mexico) and 50 g of refined cane sugar (Zulka; Zucarmex, S.A. de C.V., Culiacán, Sinaloa, Mexico). The ingredients were incorporated in a 2:1:1 ratio by weight, producing a 200-g batch containing 50% laboratory chow, 25% butter, and 25% cane sugar (w/w). The ingredients were thoroughly mixed until a homogeneous formulation was obtained and baked at 180 °C for 20 min.

In the supplementation phase, animals received one of the following cookies: a biotransformed oat and black bean cookie, formulated with bean flour obtained from *Phaseolus vulgaris* (black beans) fermented with *P. ostreatus* (2.6 mg of biomass), and a non-biotransformed cookie, prepared using the same base formulation but without fungal processing. The raw materials were sourced from a local food market in Guadalupe, Nuevo León, Mexico. The cookies were produced at the Universidad de Monterrey following previously reported formulations (11), using a 50:50 (w/w) ratio of oat flour and bean flour (fermented or non-fermented depending on the group). The formulation included flour (30%), butter (22%), egg (20%), cocoa (14%), sugar (7%), sucralose (4%), baking powder (2%), and cinnamon (1%). The dough was homogenized and baked at 180 °C for 30 min under controlled conditions. The nutritional composition of diets and cookies is summarized in Table 2.

**Table 2:** Composition of base diets and supplementation cookies.

Diet	Base diet	Supplementation		
	Standard	Hypercaloric	B cookie	NB cookie
kcal/g	4.75	7.35	4.87	5.5
Carbohydrates (%)	58	40	56	50
Protein (%)	28	8	17	20
Lipids (%)	14	52	27	30
Fiber (g)	5.3	5.3	9	6.41

B, biotransformed cookie; NB, non-biotransformed cookie; kcal, kilocalories; g, grams; mg, milligrams.

### 2.3 Biochemical parameters

Glucose levels were measured in blood samples obtained after a 12-h fast at weeks 4, 12, and 20. Baseline (week 0) blood sampling was not performed because mice were recently weaned juveniles. Sampling was minimized at this stage to reduce stress and preserve welfare. Capillary blood samples were collected from the tail tip via puncture for glucose determination, following a minimally 12-h fast invasive approach consistent with established guidelines for blood sampling in mice (51). Glucose levels were subsequently measured using a glucometer with compatible test strips (Accu-Chek® Performa; Roche Diagnostics, Mannheim, Germany) (16). Triglyceride levels were assessed at weeks 12 and 20 using the Accutrend® Plus lipid meter (Hoffmann-La Roche) with reactive strips. An oral glucose tolerance test (OGTT) was performed at weeks 4 and 20 following standard procedures for murine metabolic testing (16, 17). Blood glucose levels were measured at baseline (0 min) and at 15, 30, 45, 60, and 120 min after oral glucose administration.

### 2.4 Tissue collection

At the time of sacrifice, liver, whole bowel, and inguinal, mesenteric, and retroperitoneal adipose depots were excised using standardized anatomical landmarks and weighed using an internally calibrated sensor analytical balance (LB-42AWH; Labotronics Scientific, Wilmington, DE, USA; resolution: 0.01 g), following general recommendations for organ-weight assessment (18). Relative tissue weight was calculated as tissue weight divided by final body weight and expressed as a percentage. After weighing, tissues were rinsed with saline and fixed in 10% neutral-buffered formalin.

### 2.5 Inflammatory markers

Whole blood was allowed to clot and was then centrifuged. The separated serum was stored at −70 °C until analysis. Serum IL-6, IL-10, and TNF-*α* concentrations were determined using mouse-specific enzyme-linked immunosorbent assay kits: Mouse IL-6 ELISA Kit (KMC0061), Mouse IL-10 ELISA Kit (BMS614), and Mouse TNF-α High Sensitivity ELISA Kit (BMS607-2HS) (Thermo Fisher Scientific, Waltham, MA, USA). Assays were performed according to the manufacturer’s instructions, and samples were analyzed in triplicate using 50 μL of serum per well. Absorbance was measured at 450 nm using a Synergy H1 Multi-Mode Reader operated with Gen5 software (BioTek Instruments, Winooski, VT, USA). Cytokine concentrations were calculated using four-parameter logistic standard curves.

### 2.6 Statistical analysis

Data analysis was conducted using IBM SPSS Statistics version 25 and GraphPad Prism version 8. Normality and homogeneity of variance were assessed using the Shapiro–Wilk and Levene tests, respectively. Parametric data were analyzed with one-way or repeated-measures ANOVA followed by Tukey’s *post hoc* test or Student’s *t*-test, as appropriate. Non-parametric data were evaluated using the Mann–Whitney U test. Correlations were assessed using Pearson’s correlation coefficient. To facilitate the integrated interpretation of metabolic, tissue, and inflammatory outcomes across the nine experimental groups, a heatmap was generated using standardized group mean values (z-scores) for selected variables (body weight, fasting glucose, OGTT peak glucose, liver ratio, adipose ratio, IL-6, TNF-*α*, and IL-10). Color intensity represented relative magnitude, where warmer colors indicate higher values and cooler colors indicate lower values. Differences were considered statistically significant at *p <* 0.05 (*p < 0.05; **p < 0.01; ***p < 0.001).

## 3 Results

### 3.1 Phase 1

#### 3.1.1 Diet-related changes in body weight and food intake

In Phase 1, mice were assigned to either a hypercaloric or standard diet group, and data were analyzed accordingly.

**Figure 2:**
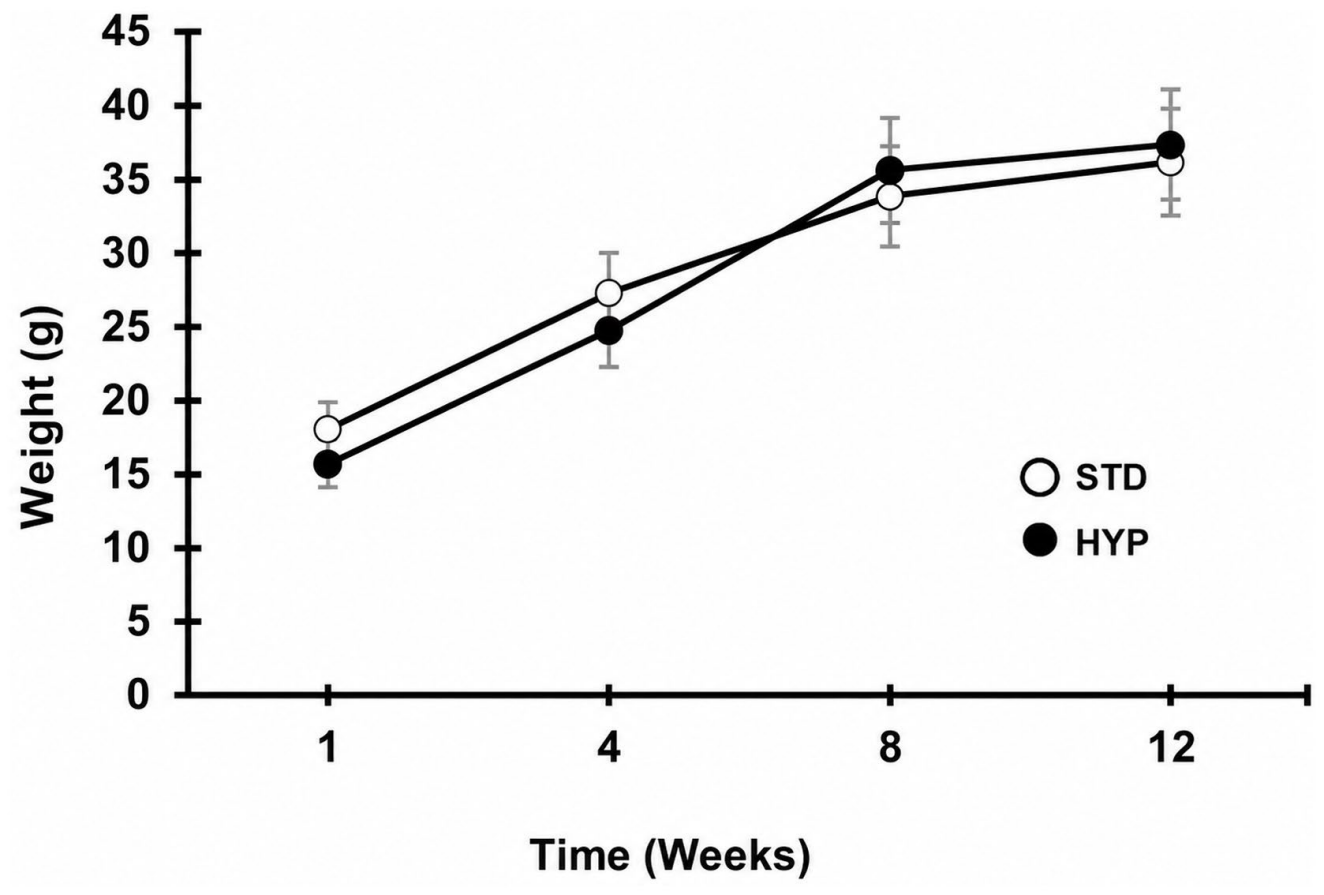
Body weight evolution during Phase 1 (weeks 1–12). Data are presented as mean ± SEM. Groups represent pooled data from all subgroups within each dietary condition (STD, *n* = 15; HYP, *n* = 30). Statistical analysis was performed using repeated measures ANOVA.

Body weight did not differ significantly between the STD and HYP groups at weeks 1, 4, 8, or 12 (Figure 2).

During Phase 1, total food intake increased between weeks 1 and 4 in both dietary groups. The mean increase was 2.48 g/day in the STD group and 3.45 g/day in the HYP group (*p* < 0.001 for both comparisons). No significant differences in total food intake were observed between the STD and HYP groups at weeks 1, 4, 8, or 12 (*p* > 0.05). Nevertheless, nutrient intake differed according to dietary condition. Protein intake was higher in the STD group at all evaluated time points (*p* < 0.001), whereas lipid intake was higher in the HYP group at weeks 1, 4, 8, and 12 (*p* ≤ 0.02). Energy intake was higher in the HYP group at weeks 1 and 4 (*p* = 0.04 and p < 0.001, respectively), but the difference was not significant at weeks 8 or 12 (Table 3).

**Table 3:** Distribution of macronutrients in total intake during Phase 1 by groups.

Variable	Week 1			Week 4			Week 8			Week 12		
	STD	HYP	*p*	STD	HYP	*p*	STD	HYP	*p*	STD	HYP	*p*
Intake (g)	2.44	2.25	0.57	5.22	5.70	0.10	5.31	5.24	0.95	5.91	5.64	0.77
kcal	11.57	16.54	0.04*	24.81	41.89	0.001**	25.23	38.49	0.18	28.07	41.49	0.07
Carbohydrates (g)	1.41	0.90	0.01*	3.03	2.28	0.46	3.08	2.09	0.09	3.43	2.26	0.02*
Protein (g)	0.68	0.18	0.001**	1.46	0.46	0.001**	1.49	0.42	0.001**	1.65	0.45	0.001**
Lipids (g)	0.34	1.17	0.001**	0.73	2.96	0.001**	0.74	2.72	0.02*	0.83	2.94	0.001**
Fibre (g)	0.13	0.12	0.80	0.27	0.30	0.10	0.28	0.28	0.95	0.31	0.30	0.77

The results are expressed as mean ± SEM. Student’s *t*-test was used for statistical analysis to compare groups from week 1 to week 12. STD, standard diet; HYP, hypercaloric diet. * = *p* < 0.05, **= *p* < 0.005.

Although triglyceride concentrations, fasting glucose concentrations, and oral glucose tolerance were also assessed, no significant between-group differences were observed. These results are presented in the supplementary materials (Supplementary Figures S1, S2).

### 3.2 Phase 2

#### 3.2.1 Supplementation-related changes in body weight and food intake

**Figure 3:**
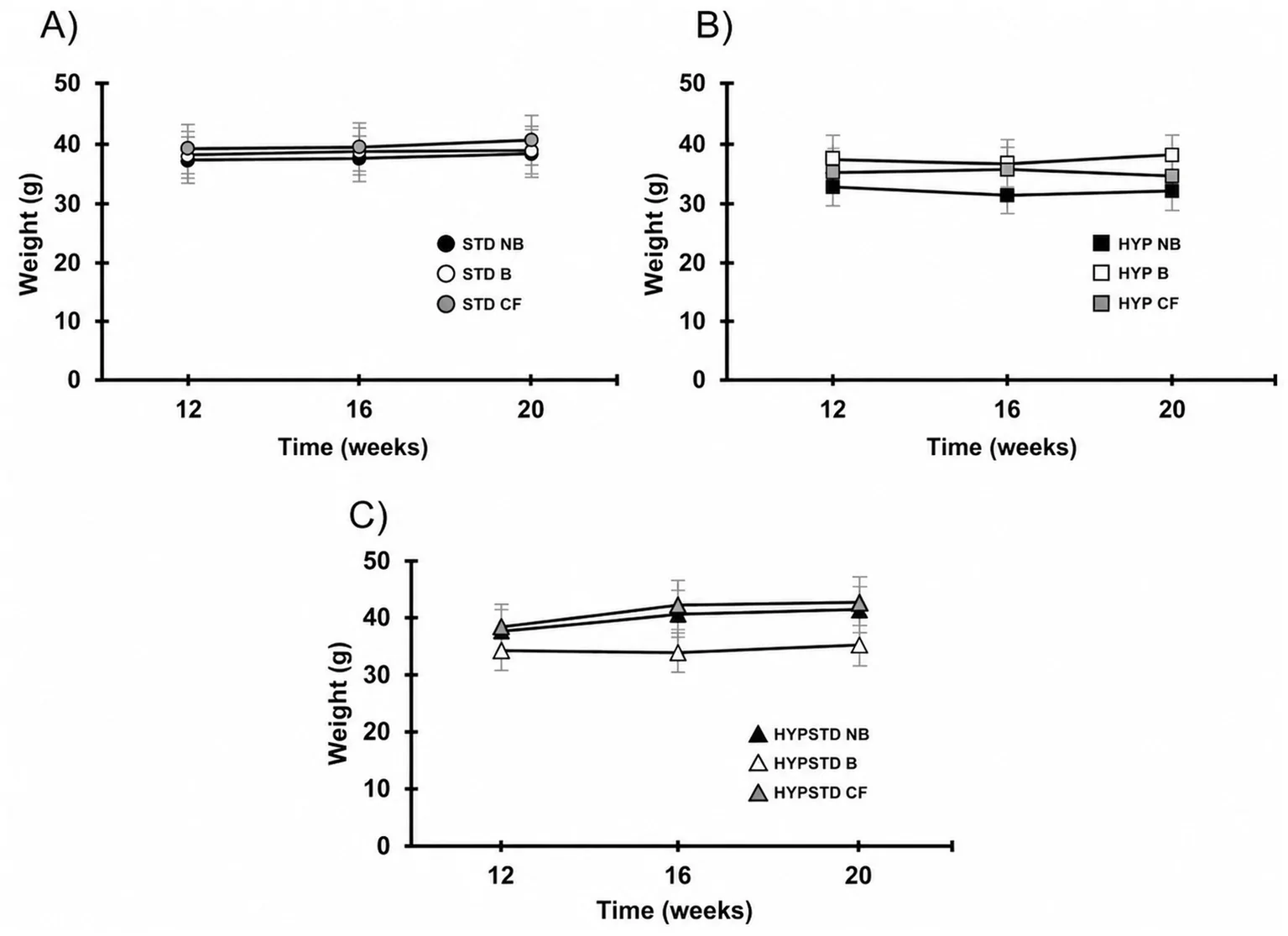
Body weight changes during Phase 2 (weeks 12–20). Data are presented as mean ± SEM. **(A)** Standard diet groups (STD NB, STD B, STD CF), **(B)** hypercaloric diet groups (HYP NB, HYP B, HYP CF), and **(C)** hypercaloric-to-standard diet groups (HYPSTD NB, HYPSTD B, HYPSTD CF). Differences were analyzed using one-way ANOVA followed by Tukey’s *post hoc* test (*n* = 5 per group).

In the STD groups, body weight remained comparable among treatments at weeks 12, 16, and 20 (*p =* 0.341, *p =* 0.366, and *p =* 0.181, respectively). In the HYP groups, the HYP NB group exhibited lower body weight than the HYP B group at week 12 (*p =* 0.046), week 16 (*p =* 0.005), and week 20 (*p <* 0.004). In the HYPSTD groups, the HYPSTD B group showed lower body weight than the HYPSTD NB group at week 12 (*p =* 0.033), whereas no significant differences were detected at weeks 16 or 20 (*p* = 0.093 and *p =* 0.464, respectively). In contrast, body weight in the HYPSTD B group remained lower than the HYPSTD CF group at weeks 12, 16, and 20 (*p =* 0.041, *p =* 0.006, and *p =* 0.011, respectively) (Figure 3).

In the STD groups, intake differed at week 16 (*p <* 0.001), where the STD B group showed the lowest total intake (4.124 g), while the STD CF group presented the highest value (11.36 g); the STD NB group showed intermediate intake (6.905 g). In the HYP groups, no differences were observed in total intake at week 16 (*p =* 0.707), although significant differences were detected for energy and macronutrient intake (*p =* 0.007 for all variables), with numerically higher values in the HYP NB group. At week 20, the HYP groups differed in total intake and nutrient composition (*p =* 0.007), mainly due to the higher intake observed in the CF group compared with supplemented groups (Table 4).

**Table 4:** Intake composition by group.

Week	Group	Intake (g)	kcal	CHO (g)	PRO (g)	FAT (g)
12	STD NB	10.872	52.323^ac^	5.384^ac^	2.856^ac^	2.630
	STD B	11.033	53.093^bc^	5.464^bc^	2.898^bc^	2.669^bc^
	STD CF	10.940	51.965^c^	5.418^c^	2.874^c^	2.646^c^
	*p*	0.890	<0.001**	<0.001**	<0.001**	<0.001**
	HYP NB	10.434^ac^	63.645^ab^	4.150^ac^	0.811^ac^	5.465^ac^
	HYP B	11.284^bc^	68.067^ba^	4.488^bc^	0.877^bc^	5.911^bc^
	HYP CF	8.976^c^	65.974	3.570^c^	0.698^c^	4.702^c^
	*p*	<0.001**	<0.001**	<0.001**	<0.001**	<0.01*
	HYPSTD NB	9.309	44.869^ac^	3.702^ac^	0.723^ac^	4.876^ac^
	HYPSTD B	9.301	44.84^bc^	3.699^bc^	0.723^bc^	4.871^bc^
	HYPSTD CF	11.112	52.782^c^	4.420^c^	0.864^c^	5.820^c^
	*p*	0.114	0.015*	0.017*	0.017*	0.017*
16	STD NB	6.905^a b^	33.260^ab^	3.420^ab^	1.814	1.670
	STD B	4.124^cb^	19.883^bac^	2.042^ba^	1.084^bc^	1.098^bc^
	STD CF	11.36^b^	53.960^cb^	5.626^cb^	2.984^c^	2.748^c^
	*p*	<0.001**	<0.001**	<0.001**	<0.001**	<0.001**
	HYP NB	4.864	27.904	1.934^ab^	0.378^ab^	2.548^ab^
	HYP B	4.458	25.636	1.772^ba^	0.346^ba^	2.335^ba^
	HYP CF	4.706	34.589	1.872	0.366	2.464
	*p*	0.707	0.007	0.007*	0.007*	0.007*
	HYPSTD NB	4.216	20.403	1.676	0.327	2.209
	HYPSTD B	4.196	20.269	1.669	0.326	2.198
	HYPSTD CF	4.860	23.668	1.933	0.378	2.546
	*p*	0.701	0.687	0.689	0.693	0.697
20	STD NB	5.351	25.804	2.650	1.406	1.295
	STD B	4.734	22.841	2.344	1.244	1.146
	STD CF	9.468	44.973	4.688	2.488	2.290
	*p*	0.441	0.430	0.421	0.414	0.470
	HYP NB	4.690^ac^	27.280^ac^	1.865^ac^	0.364^ab^	2.457^ab^
	HYP B	4.587^bc^	27.862^bc^	1.825^bc^	0.356^bc^	2.402^bc^
	HYP CF	6.676^c^	49.069^c^	2.726^c^	0.532^c^	3.590^c^
	*p*	0.007 *	0.007*	0.006*	0.007*	0.007*
	HYPSTD NB	5.498	26.500	2.187	0.427	2.880
	HYPSTD B	5.906	28.433	2.349	0.459	3.094
	HYPSTD CF	6.672	32.480	2.689	0.525	3.542
	*p*	0.507	0.486	0.508	0.544	0.503

Presented by week and supplemented group. Data expressed as mean ± SEM. Differences between groups were analyzed using one-way ANOVA and Tukey’s *post hoc* test. a = ac = NB vs. CF, bc = B vs. CF, ab = NB vs. B. *n* = 5 per group. **p < 0.005, *p < 0.05. STD NB, standard non-biotransformed cookie; STD B, standard biotranformed cookie; STD CF, standard cookie-free; HYP NB, hypercaloric non-biotransformed cookie; HYP B, hypercaloric biotranformed cookie; HYP CF, hypercaloric cookie-free; HYPSTD NB, hypercaloric-standard non-biotransformed cookie; HYPSTD B, hypercaloric-standard biotranformed cookie; HYPSTD CF, hypercaloric-standard cookie-free. ** = *p* < 0.001, * = *p* < 0.01.

A progressive change in consumption patterns was observed across Phase 2 (Table 1). At week 12, no significant differences were observed between diet and cookie intake across any group (*p >* 0.05), indicating comparable initial consumption patterns.

By week 16, significant differences emerged in several groups. Increased cookie consumption relative to diet intake was observed in the HYP NB (*p <* 0.001), HYP B (*p <* 0.004), HYPSTD NB (*p <* 0.001), and HYPSTD B (*p <* 0.001) groups, suggesting a shift in preference toward the cookie in hypercaloric conditions and supplemented groups.

At week 20, significant differences were observed in the STD NB (*p =* 0.02), STD B (*p =* 0.01), and HYP NB (*p =* 0.02) groups, where cookie consumption remained higher than diet intake. In contrast, no significant differences were found in the HYP B (*p =* 0.83), HYPSTD NB (*p =* 0.11), and HYPSTD B (*p =* 0.88) groups, indicating a more balanced or variable consumption pattern at this stage.

#### 3.2.2 Supplementation-related changes in biochemical parameters

**Figure 4:**
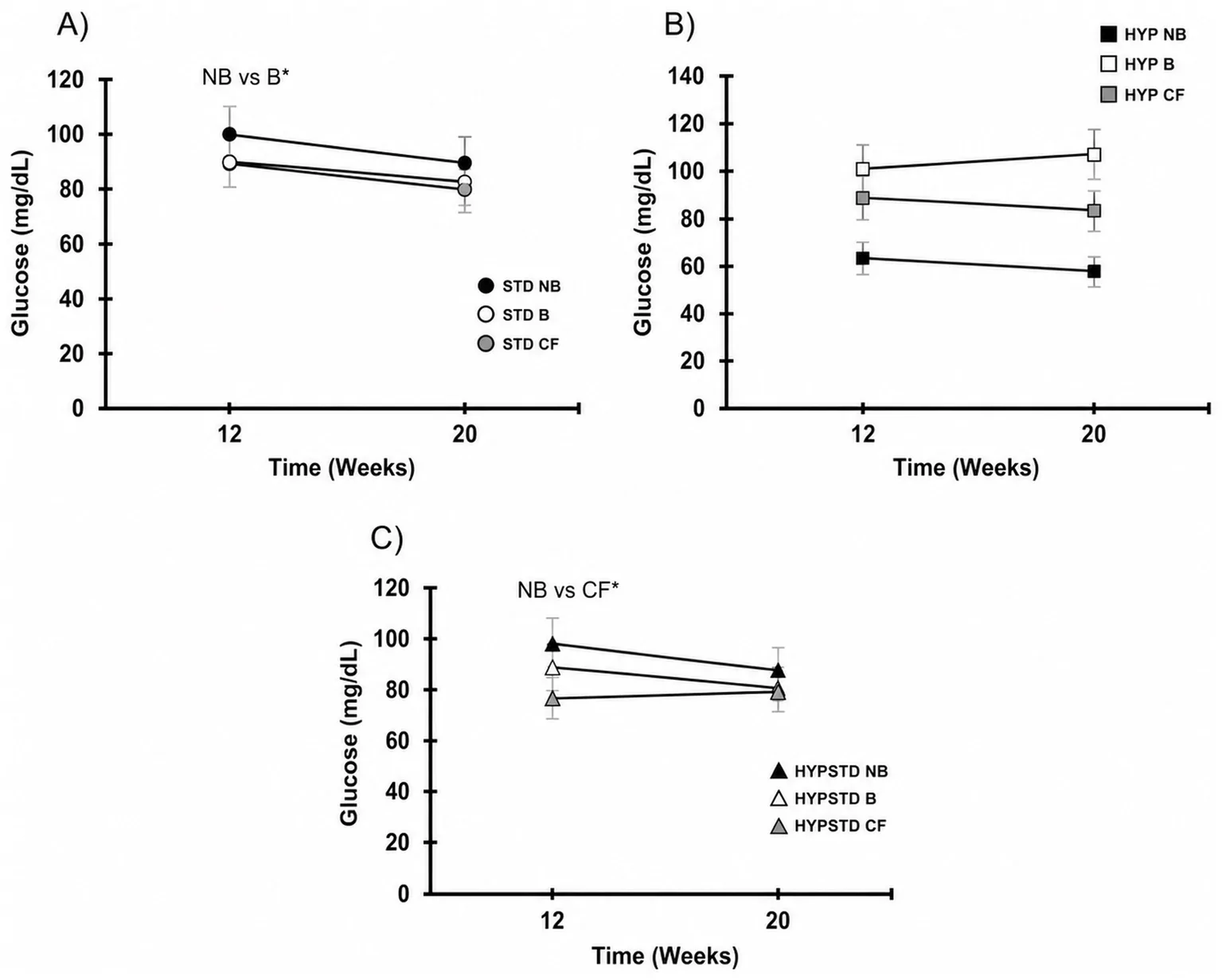
Fasting glucose levels during Phase 2 (weeks 12 and 20). Data are presented as mean ± SEM. **(A)** Standard diet groups, **(B)** hypercaloric diet groups, and **(C)** hypercaloric-to-standard diet groups. Statistical comparisons between groups are indicated (*p < 0.05) using one-way ANOVA followed by Tukey’s *post hoc* test (*n* = 5 per group).

Overall fasting glucose levels were comparable among experimental groups at weeks 12 and 20, although significant pairwise differences were detected in specific comparisons at week 12. In the STD groups, the STD NB group showed higher glucose levels than the STD B group at week 12 (99 vs. 89 mg/dL; *p =* 0.022). The same numerical pattern remained at week 20, although differences were no longer significant (*p =* 0.928). In the HYP groups, no significant differences were observed between the HYP NB and HYP B groups at either time point (*p =* 0.229 and *p =* 0.387), although the HYP B group showed numerically higher glucose values than the HYP NB group. In the HYPSTD groups, the HYPSTD NB group presented higher glucose levels than the HYPSTD CF group at week 12 (98 vs. 78 mg/dL; *p =* 0.040), but this difference was not maintained at week 20 (*p =* 0.456) (Figure 4).

**Figure 5:**
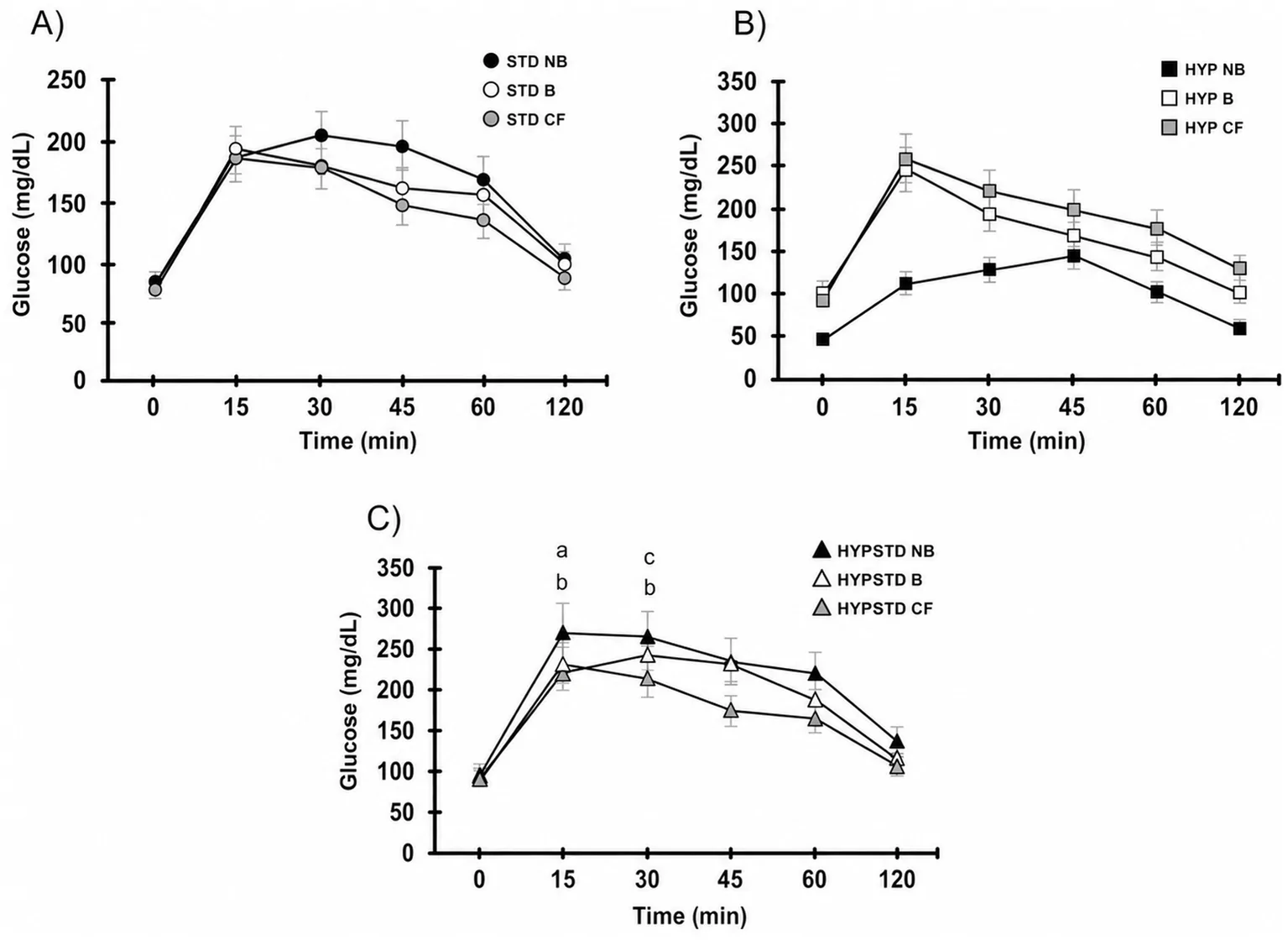
Oral glucose tolerance test (OGTT) curves during phase 2 (week 20). Blood glucose levels were measured at 0, 15, 30, 45, 60, and 120 min. Data are presented as mean ± SEM. **(A)** Standard diet groups, **(B)** hypercaloric diet groups, and **(C)** hypercaloric-to-standard diet groups. Different letters indicate statistically significant differences between groups (*p* < 0.05), analyzed using one-way ANOVA followed by Tukey’s *post hoc* test (*n* = 5 per group).

During the oral glucose tolerance test, the STD groups showed comparable glucose responses across time points. The STD NB group presented values similar to STD B and STD CF groups at minutes 15, 30, and 45 (minute 15: *p =* 0.951 and *p =* 0.151; minute 30: *p =* 0.798 and *p* = 0.816; minute 45: *p =* 0.146 and 0.920, respectively). In the HYP groups, no significant differences were detected between the HYP NB group and HYP B or HYP CF groups from minute 0 to 120, although the HYP NB group showed numerically lower glucose values throughout most of the test (*p =* 0.188, *p =* 0.072, *p =* 0.606, *p =* 0.430, and *p =* 0.248). In the HYPSTD groups, a significant difference was observed at minute 15, where the HYPSTD NB group showed higher glucose levels than the HYPSTD CF and HYPSTD B groups (262 vs. 215 and 224 mg/dL; *p <* 0.05 and *p =* 0.022, respectively). At minute 30, the HYPSTD NB group maintained higher glucose levels only compared with the HYPSTD CF group (258 vs. 224 mg/dL; *p =* 0.002) (Figure 5).

#### 3.2.3 Supplementation-related changes in relative tissue weights

**Figure 6:**
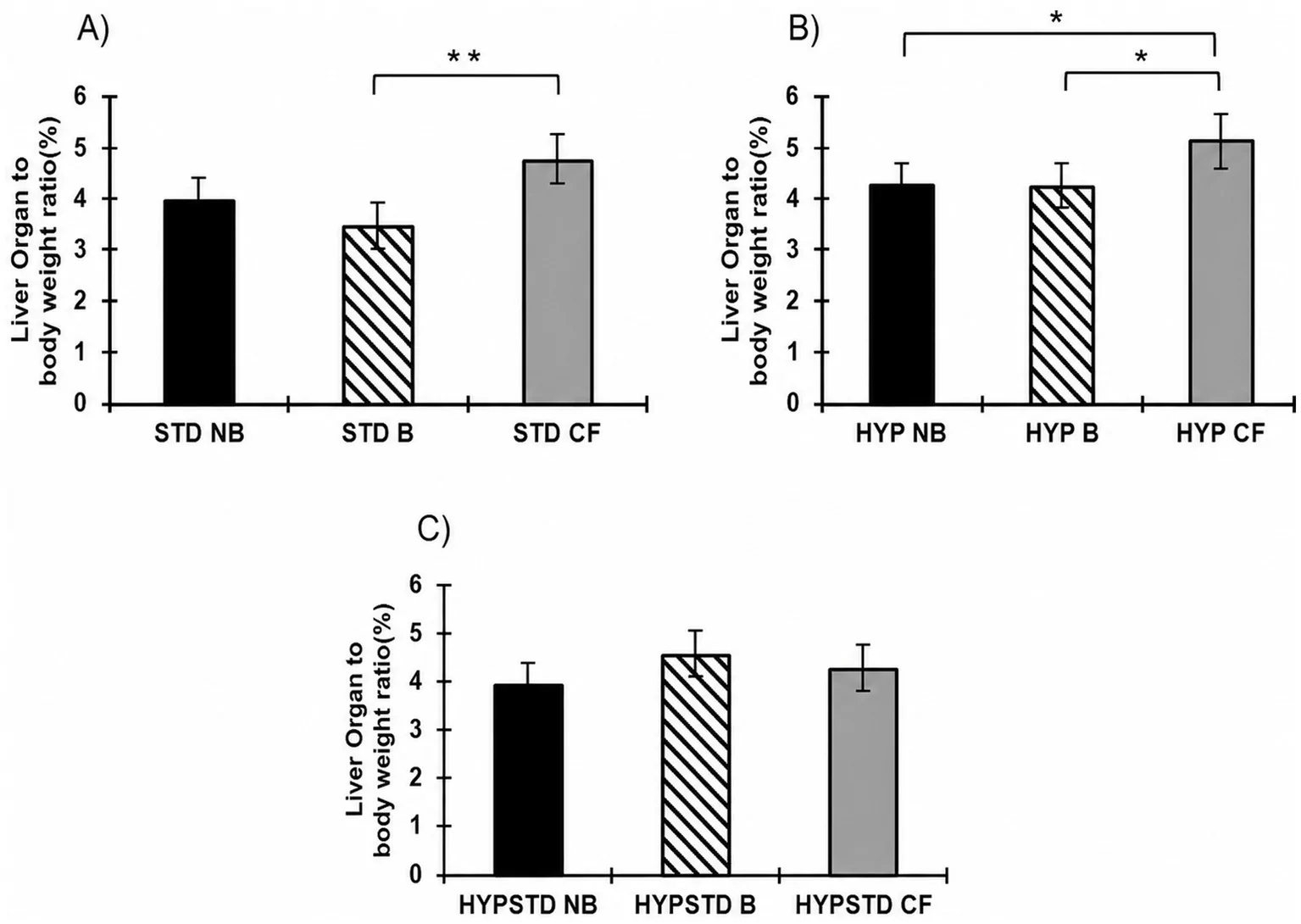
Liver organ-to-body weight ratio (%) during Phase 2. Data are presented as mean ± SEM. **(A)** Standard diet groups, **(B)** hypercaloric diet groups, and **(C)** hypercaloric-to-standard diet groups. Asterisks indicate statistically significant differences between groups (*p < 0.05; **p < 0.01) using one-way ANOVA followed by Tukey’s *post hoc* test (*n* = 5 per group).

For the liver organ-to-body weight ratio, in the STD groups, the STD CF group showed a significantly higher ratio than STD B (4.6% vs. 3.4%; *p =* 0.001), whereas no significant differences were observed between STD CF and STD NB groups. In the HYP groups, the cookie-free group also showed a higher liver ratio than both supplemented groups (HYP NB and HYP B) (5.0% vs. 4.2%; *p =* 0.046). No significant differences were observed among the HYPSTD groups (Figure 6).

**Figure 7:**
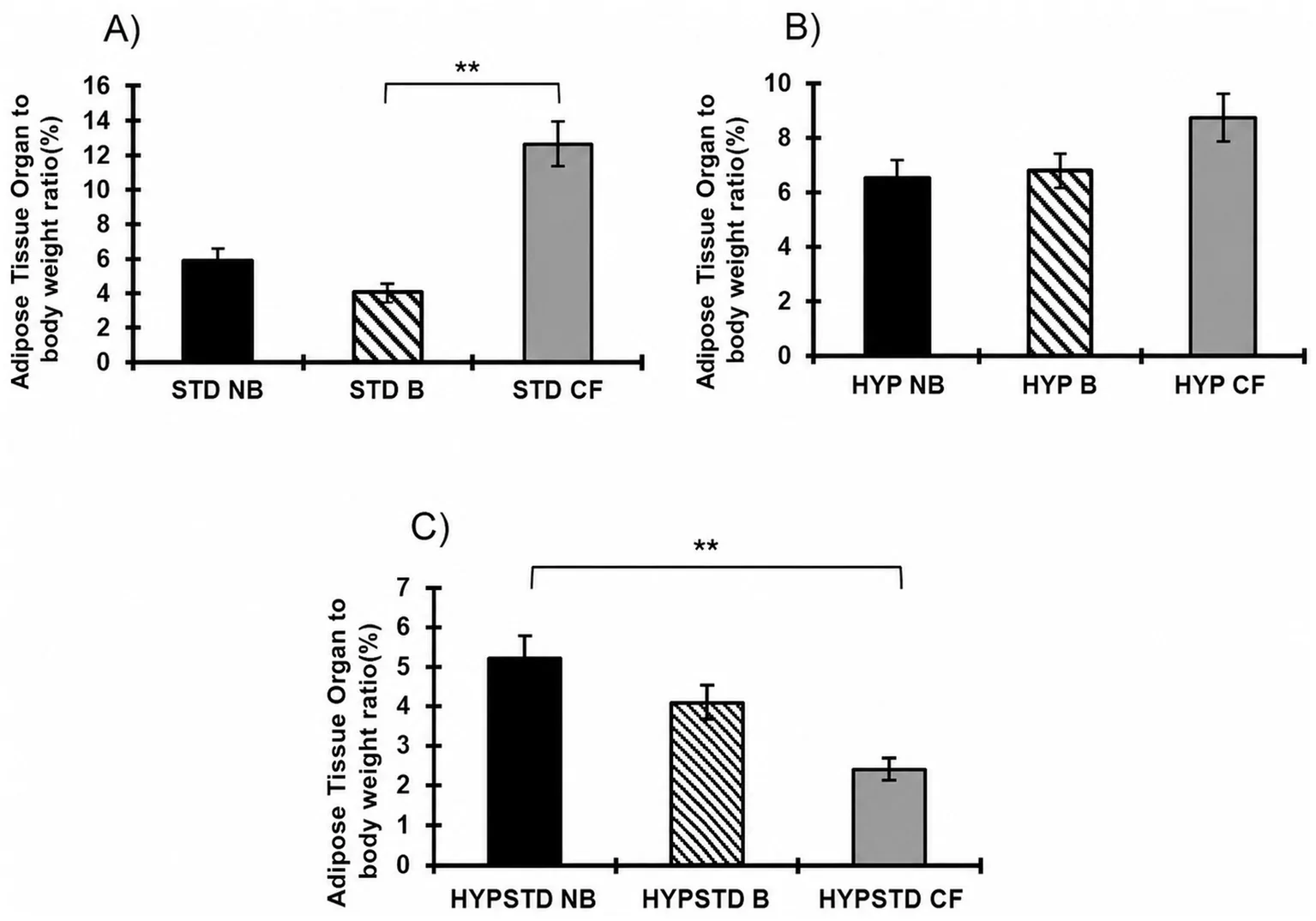
Adipose tissue organ-to-body weight ratio (%) during Phase 2. Data are presented as mean ± SEM. **(A)** Standard diet groups, **(B)** hypercaloric diet groups, and **(C)** hypercaloric-to-standard diet groups. Asterisks indicate statistically significant differences between groups (*p < 0.05; **p < 0.01) using one-way ANOVA followed by Tukey’s *post hoc* test (*n* = 5 per group).

In the adipose tissue organ-to-body weight ratio, significant differences were observed in the STD groups, where STD CF showed a higher ratio than STD B (12.5% vs. 4.1%; *p =* 0.019). In the HYP groups, no significant differences were found among treatments, although the HYP CF group showed numerically higher values than HYP NB and HYP B (*p =* 0.661 and *p =* 0.808). In the HYPSTD groups, the cookie-free group showed a lower adipose tissue ratio than the HYPSTD NB group (2.3% vs. 5.2%; *p =* 0.01), while the HYPSTD B group showed intermediate values (Figure 7).

**Figure 8:**
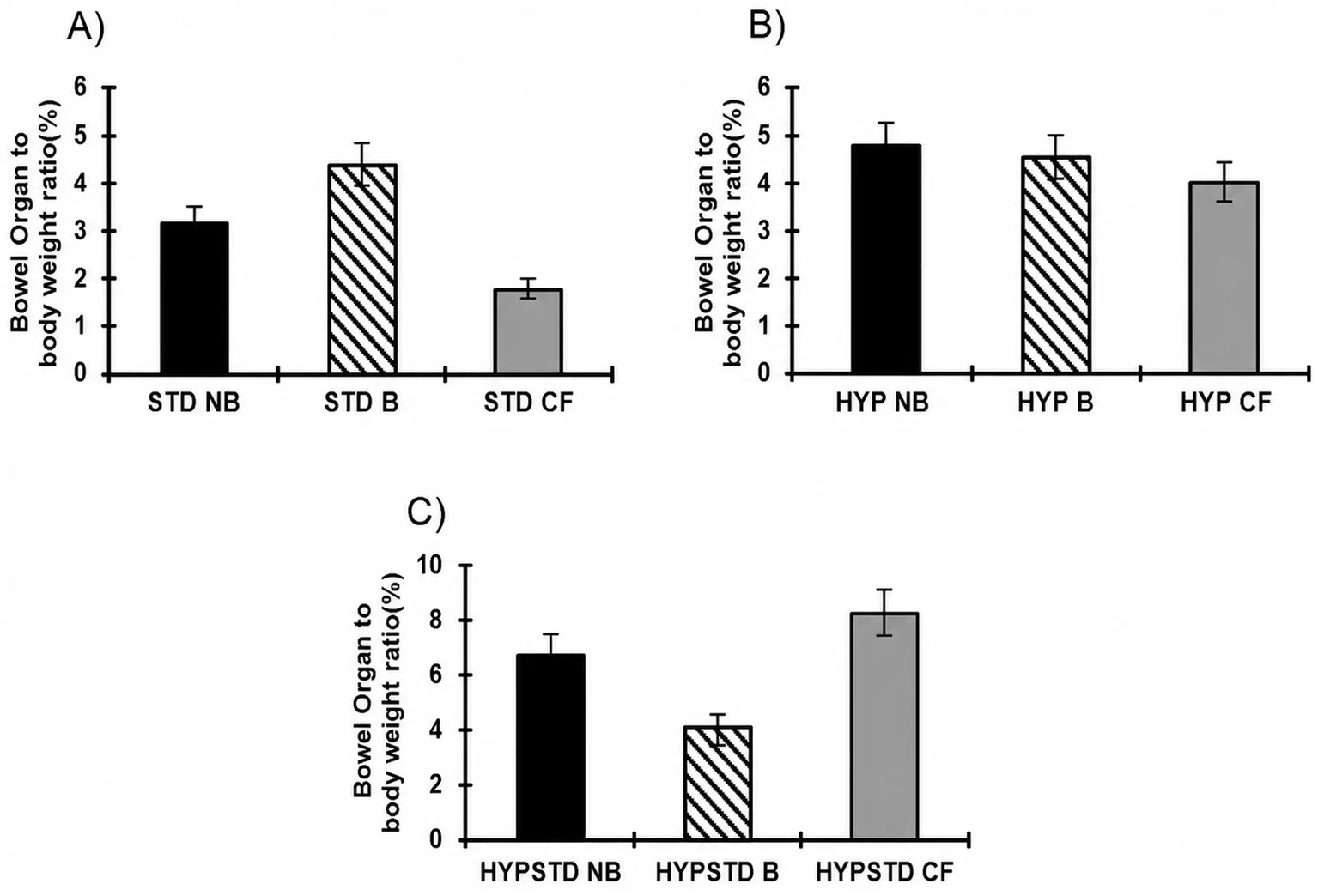
Bowel organ-to-body weight ratio (%) during Phase 2. Data are presented as mean ± SEM. **(A)** Standard diet groups, **(B)** hypercaloric diet groups, and **(C)** hypercaloric-to-standard diet groups. Differences between groups were analyzed using one-way ANOVA followed by Tukey’s *post hoc* test (*n* = 5 per group). No significant differences were observed between groups.

In the STD groups, bowel organ-to-body weight ratio was similar among treatments, although the STD B group showed numerically higher values and STD CF group the lowest values (4.4, 3.2, and 1.7% for STD B, STD NB, and STD CF, respectively; *p =* 0.078, 0.878, and 0.175). In the HYP groups, all three treatments also showed comparable bowel ratios, with values ranging from 3.9 to 4.7% and no significant differences between groups (*p =* 0.453, 0.989, and 0.380). In the HYPSTD groups, the highest bowel ratio was observed in the HYPSTD CF group, followed by the HYPSTD NB group, whereas the HYPSTD B group showed the lowest value; however, these differences were not statistically significant (8.1, 6.6, and 4.1%, respectively; *p =* 0.346, 0.648, and 0.799) (Figure 8).

#### 3.2.4 Supplementation-related changes in inflammatory markers

**Figure 9:**
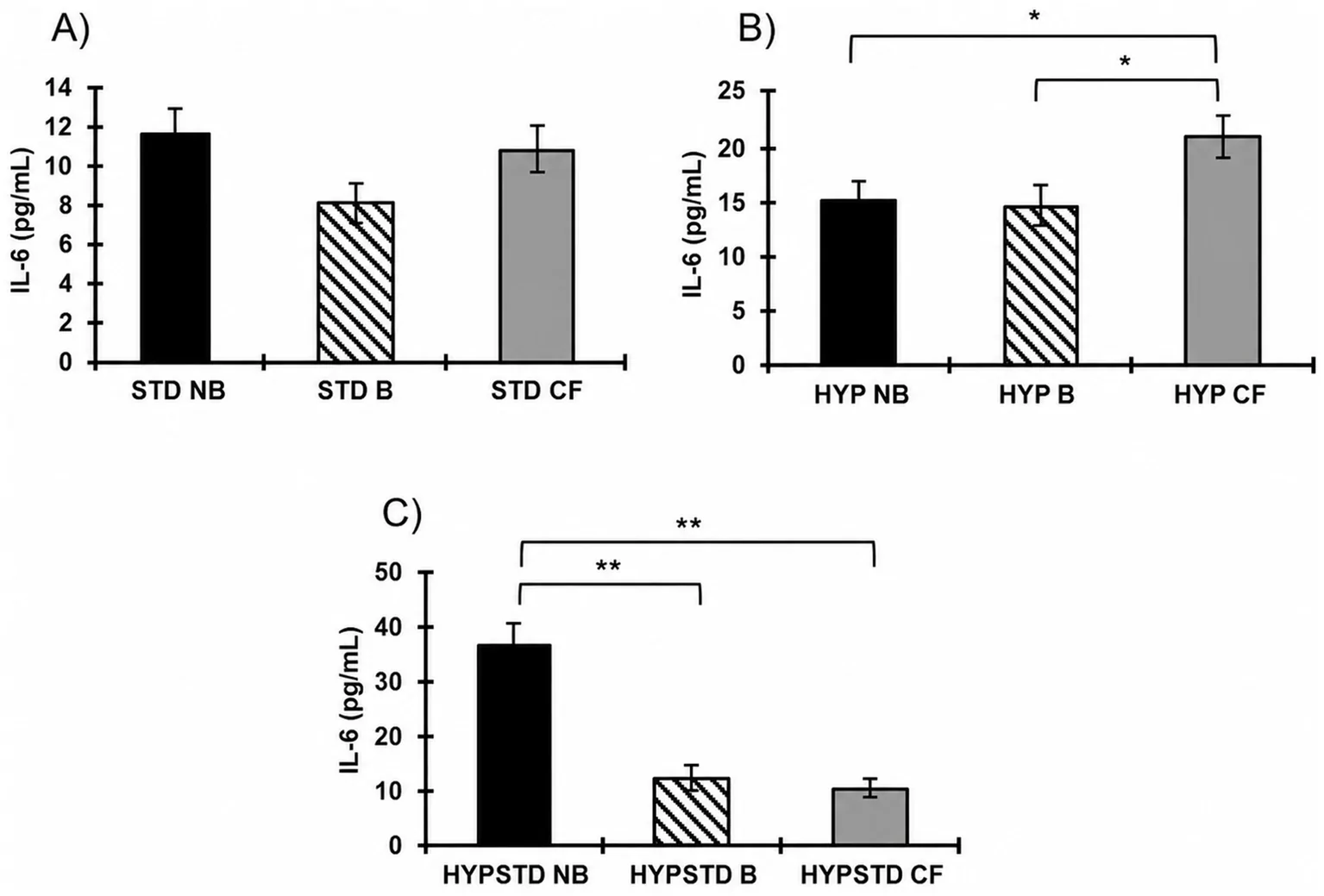
Serum IL-6 levels during Phase 2. Data are presented as mean ± SEM (*n* = 3 per group). **(A)** Standard diet groups, **(B)** hypercaloric diet groups, and **(C)** hypercaloric-to-standard diet groups. Differences between groups were analyzed using one-way ANOVA followed by Tukey’s *post hoc* test. Asterisks indicate statistically significant differences between the indicated groups (*p < 0.05; **p < 0.01).

In the STD condition, serum IL-6 concentrations did not differ significantly among treatments, although the mean concentration was numerically lower in STD B mice than in STD NB and STD CF mice (7.4 versus 11.3 and 10.4 pg./mL, respectively; *p* = 0.178 and *p* = 0.317). In the HYP condition, IL-6 concentrations were lower in HYP NB and HYP B mice than in HYP CF mice (15.0 and 14.8 versus 20.3 pg./mL, respectively; *p* < 0.05). In the HYPSTD condition, IL-6 was higher in HYPSTD NB mice than in HYPSTD B and HYPSTD CF mice (36 versus 12 and 10 pg./mL, respectively; *p* < 0.005; Figure 9).

**Figure 10:**
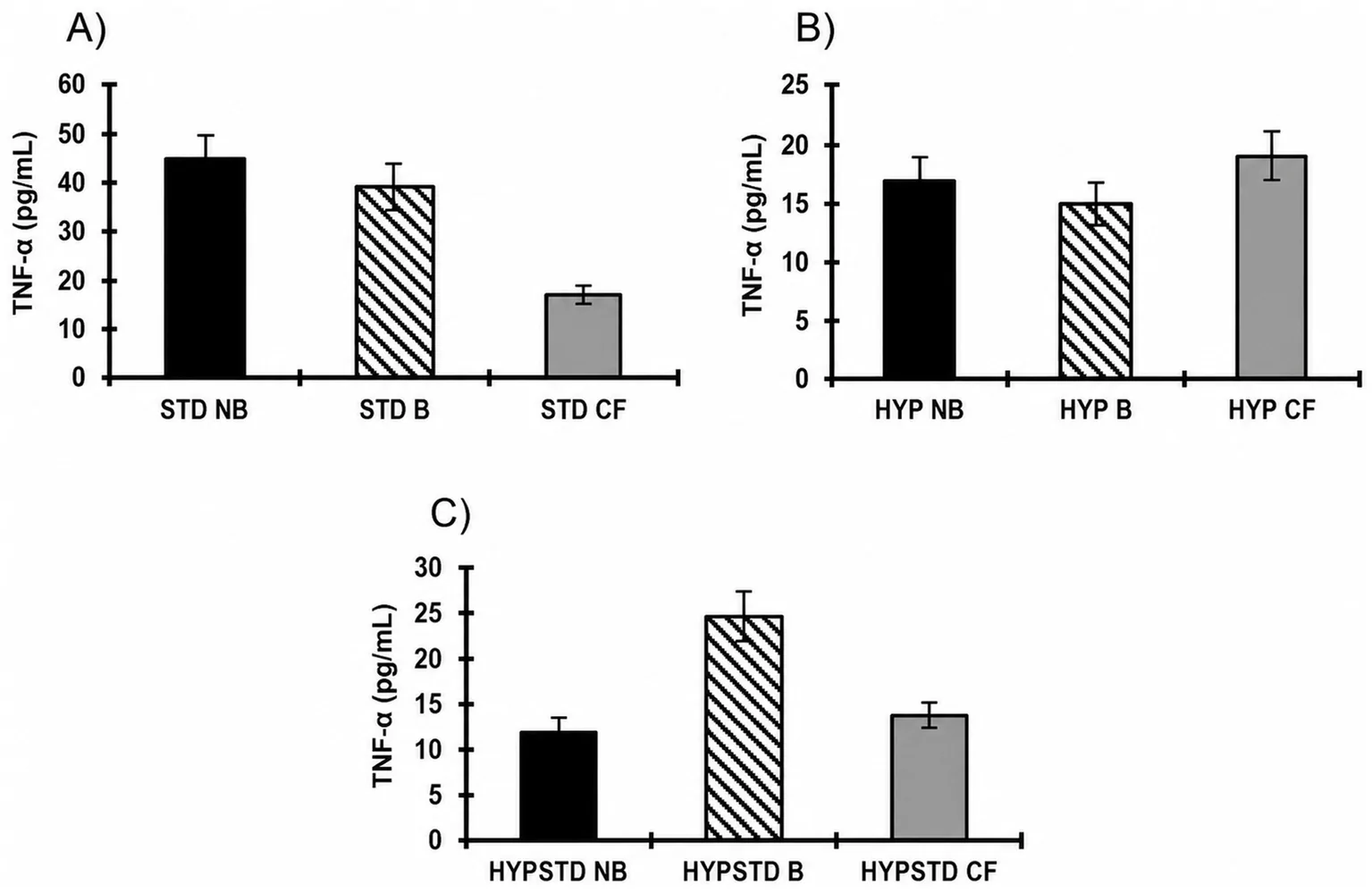
Serum TNF-α levels during Phase 2. Data are presented as mean ± SEM (*n* = 3 per group). **(A)** Standard diet groups, **(B)** hypercaloric diet groups, and **(C)** hypercaloric-to-standard diet groups. Differences between groups were analyzed using one-way ANOVA followed by Tukey’s *post hoc* test. No significant differences were observed between groups.

Similar TNF-*α* levels were observed among the STD groups, although numerically higher concentrations were detected in the supplemented groups (STD NB and STD B) compared with the STD CF group (43 and 38 vs. 16 pg./mL, respectively; *p =* 0.098 and *p =* 0.167). In the HYP groups, cytokine levels were also similar across the three groups, with the HYP B group showing a slight, non-significant trend toward lower values (14.2 pg./mL) compared with HYP NB and HYP CF groups (16.4 and 18.2 pg./mL). In the HYPSTD groups, a trend toward increased TNF-α levels was observed in the biotransformed group compared with the HYPSTD NB and HYPSTD CF groups (24 vs. 11 and 13 pg./mL, respectively; *p =* 0.056 and *p =* 0.089); however, these differences were not statistically significant (Figure 10).

**Figure 11:**
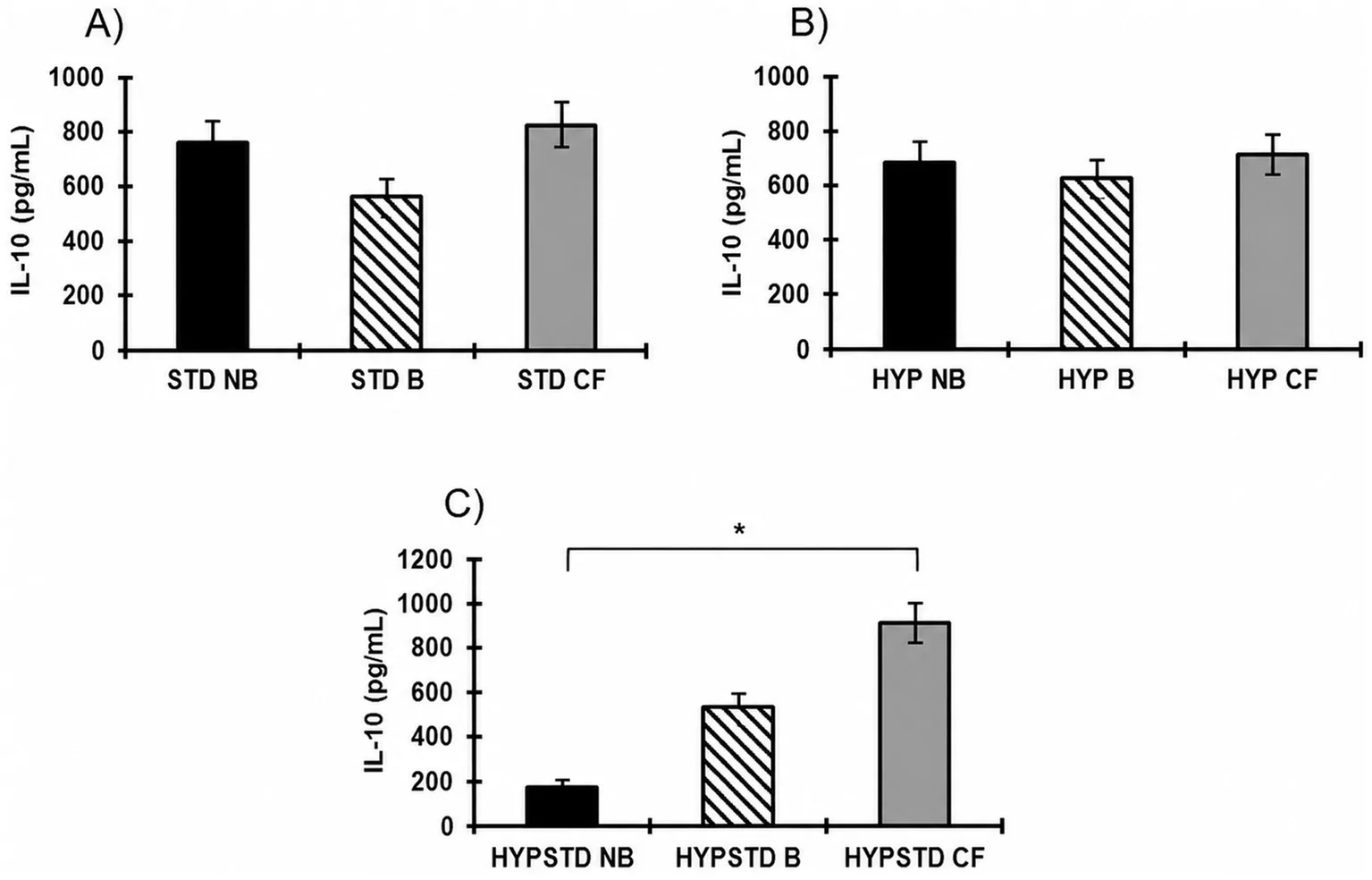
Serum IL-10 levels during Phase 2. Data are presented as mean ± SEM (*n* = 3 per group). **(A)** Standard diet groups, **(B)** hypercaloric diet groups, and **(C)** hypercaloric-to-standard diet groups. Differences between groups were analyzed using one-way ANOVA followed by Tukey’s *post hoc* test. Asterisks indicate statistically significant differences between the indicated groups (*p < 0.05).

IL-10 levels were similar in the STD groups, although the biotransformed group showed an intermediate mean concentration (553 pg./mL) compared to STD NB and STD CF groups (758 and 414 pg./mL, respectively; *p =* 0.987, 0.744). The same occurred in the HYP groups, where comparable levels were observed among groups (HYP NB vs. HYP B *p =* 0.460; HYP NB vs. HYP CF *p =* 0.732; HYP B vs. HYP CF *p =* 0.212), with mean values ranging from 610 to 700 pg./mL. In the HYPSTD groups, IL-10 levels showed a progressive increase from HYPSTD NB to HYPSTD B to HYPSTD CF groups (166, 520, and 903 pg./mL, respectively), and a significant difference was observed between the HYPSTD CF and HYPSTD NB groups, with the HYPSTD NB group showing lower levels (*p =* 0.031) (Figure 11).

#### 3.2.5 Correlation between inflammatory and biochemical parameters

**Figure 12:**
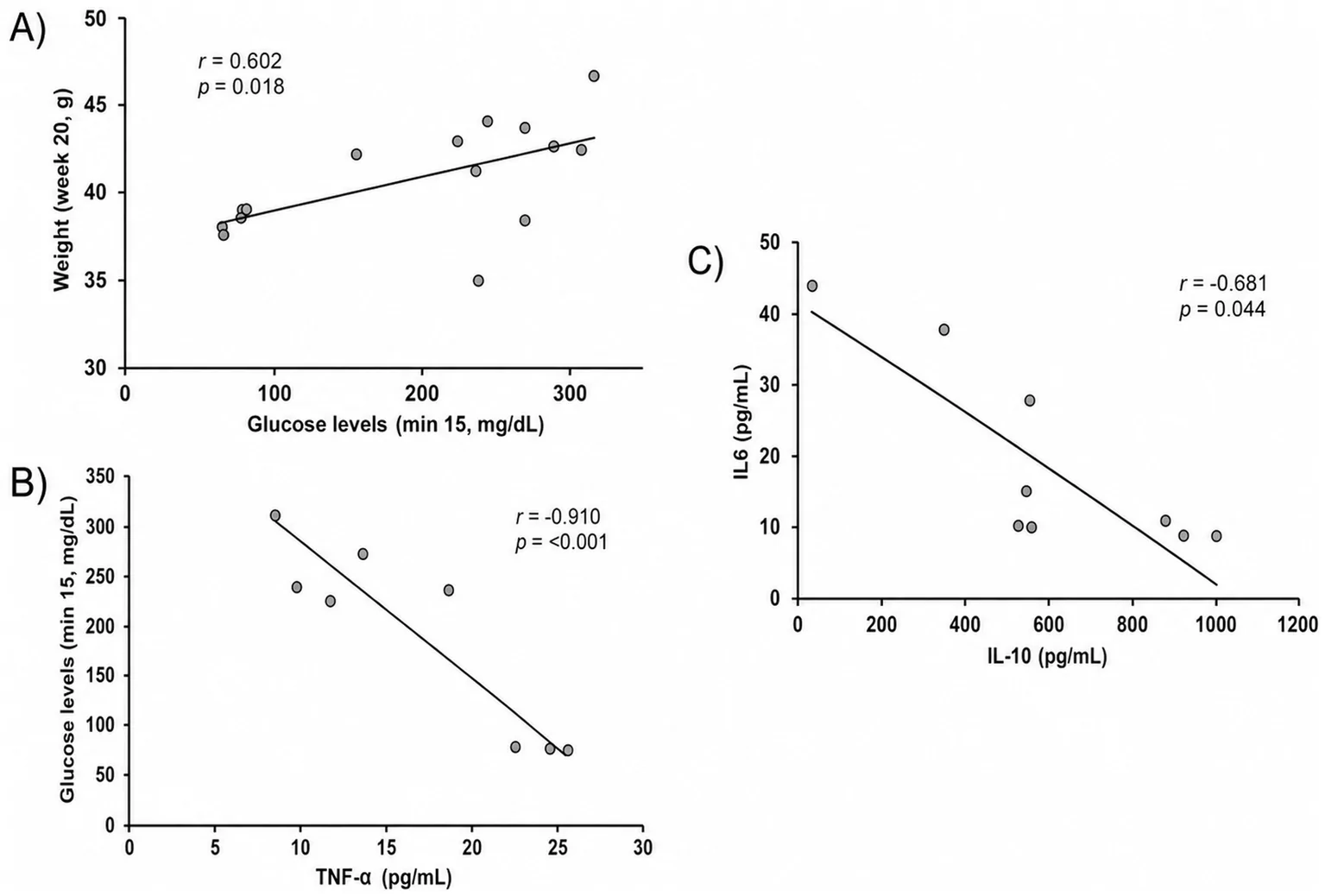
Correlation analysis using Pearson’s correlation coefficient. **(A)** Correlation between glucose levels at minute 15 during the oral glucose tolerance test (OGTT) and body weight at week 20. **(B)** Correlation between glucose levels at minute 15 during the OGTT and serum TNF-α levels. **(C)** Correlation between IL-6 and IL-10 levels. Correlation coefficients (*r*) and *p* values are indicated in each panel.

A positive correlation was observed between glucose levels at minute 15 of the oral glucose tolerance test and body weight during the final week of intervention, indicating that higher glucose levels were associated with greater body weight (*r* = 0.602, *p =* 0.018). A negative correlation was also observed between glucose levels at minute 15 of the oral glucose tolerance test and TNF-*α* levels, such that lower glucose levels were associated with higher TNF-α concentrations (*r* = −0.910, *p <* 0.001). A negative correlation was observed between IL-6 and IL-10 levels; lower IL-6 concentrations were associated with higher IL-10 concentrations. (*r* = −0.681, *p =* 0.044) (Figure 12).

#### 3.2.6 Integrated summary of metabolic, tissue, and inflammatory responses

To provide an integrated overview of the study outcomes, a heatmap based on standardized group mean values (z-scores) was generated (Supplementary Figure S3). This visualization revealed distinct response patterns among the experimental groups. The HYPSTD B group displayed relatively lower standardized OGTT peak and IL-6 values; however, the heatmap was descriptive and was not used for inferential comparisons. In contrast, the HYPSTD NB group exhibited elevated adipose tissue and IL-6 values, while the HYP CF group showed higher liver ratio values. The STD CF group presented higher IL-10 values with lower adipose tissue levels. Overall, the heatmap summarizes the differential tissue and inflammatory responses among interventions.

## 4 Discussion

Chronic systemic inflammation is a condition frequently associated with metabolic alterations induced by dietary factors. This study aimed to evaluate the effects of consuming cookies enriched with *P. ostreatus*-biotransformed flour on nutritional, tissue, and inflammatory parameters in mice with metabolic disruption induced by a high-fat diet. We hypothesized that consuming this supplemented cookie could exert a modulatory effect on nutritional, biochemical, and inflammatory markers in this supplementation model. The functional properties of the biotransformed flour used for the cookie formulation in this study were previously characterized by our research group. We have demonstrated that solid-state fermentation with *P. ostreatus* significantly increases antioxidant capacity and total polyphenol content, and improved protein digestibility in both *Phaseolus vulgaris* and *Avena sativa* (11). It is worth noting that these studies also indicated that the bioactive properties are preserved or even enhanced after simulated gastrointestinal digestion, indicating greater bioaccessibility of the functional compounds. Furthermore, the cookies made with these biotransformed flours showed greater antioxidant capacity compared to the non-fermented control, maintaining their functional properties after digestion (12). These findings support the hypothesis that fermentation with *P. ostreatus* induces structural and biochemical modifications in the food matrix that could contribute to the physiological effects observed in this *in vivo* model. However, further studies using advanced analytical techniques, such as metabolomics, are required to identify the specific compounds responsible for these effects.

During states of metabolic dysfunction, body weight and food intake are among the most responsive parameters, given that dietary composition directly influences nutrient density and distribution. Alterations in intake patterns, particularly those characterized by high proportions of sugars and fats, can disrupt carbohydrate, protein, and lipid metabolism, ultimately contributing to inflammation and the onset of non-communicable diseases (19).

The lack of significant differences in body weight during Phase 1 may be explained by the specific macronutrient composition of the hypercaloric diet. In a study by Sollano-Mendieta et al. (20), CD-1 mice fed a hypercaloric diet for 10 weeks exhibited greater weight gain compared to control groups on a standard diet. In that study, the maximum recorded weight reached 48 g, whereas in the present study, the highest body weight was 42 g. This discrepancy may be attributed to differences in the diet’s composition: Solano-Mendieta’s formulation contained 48.5% carbohydrates and 4.5% fats, while the current study employed a diet with 40% carbohydrates and 52% fats. Additionally, Guimarães et al. (21) reported that a high-carbohydrate diet was more effective than a high-fat diet in inducing metabolic alterations in mice. Another study observed that using animal-derived fats, such as lard, in a high-fat diet resulted in greater weight gain in C57BL/6 J mice after 10 weeks of intervention, compared to diets based primarily on plant-derived lipids. Mice in that group reached 33 g by week 8, consistent with the present findings in hypercaloric diet groups (22). Therefore, both differences in diet composition and the relatively short duration of the intervention may explain the limited metabolic effects observed during Phase 1.

Although total food intake was comparable between groups, the HYP group consumed more energy and lipids because of the greater energy density and fat content of the hypercaloric formulation. This distinction may help explain why tissue and inflammatory alterations appeared despite the absence of marked differences in total food mass or body weight. High-fat diets can produce metabolic alterations without a proportional increase in food weight because fat provides more energy per gram than carbohydrate or protein (23). Nevertheless, the absence of significant weight gain suggests that the duration or composition of the intervention, as well as the response of CD-1 mice, may have limited the development of a pronounced obesity phenotype.

However, during Phase 2, several parameters were altered, including body weight, oral glucose tolerance, adipose tissue accumulation, liver-to-body weight ratio, and IL-6 levels. These changes may be attributed to the cumulative intake patterns and dietary composition established during Phase 1. Specifically, the standard diet group maintained consistently high protein intake from weeks 1 to 12, whereas the hypercaloric diet group exhibited a continuous increase in fat consumption. These observations reflect the contrasting macronutrient profiles of the diets: the higher protein content of the standard diet and the higher fat content of the hypercaloric diet. Although overall body weights remained similar across groups, the HYP B group displayed a greater weight increase than the NB group, possibly due to higher intake during week 20. This coincided with a pronounced preference for the hypercaloric diet, suggesting increased lipid intake compared to the group receiving the biotransformed supplement.

The absence of changes in intake, especially among mice on a hypercaloric diet, may be attributed to the overexpression of satiety-related genes (e.g., Lepr, Lep, POMC) under conditions of overfeeding, which is commonly observed in murine models but not in humans (24). It has also been documented that suppression of these genes increases susceptibility to hypercaloric diets in mice (25).

During Phase 1, although intake levels were similar between HYP and STD groups, the macronutrient composition of the consumed food differed significantly, reflecting the respective diet formulations. Mice on the standard diet consumed more carbohydrates and proteins, consistent with their higher protein and carbohydrate content, whereas the hypercaloric groups consumed more lipids and energy overall, aligning with the energy-dense and fat-rich profile of their diet. These intake profiles likely contributed to the inflammatory and metabolic alterations observed during Phase 2.

In Phase 2, despite the absence of differences in overall macronutrient intake among the standard and hypercaloric diet groups, a distinct preference for the supplemented cookies (both biotransformed and non-biotransformed) was evident. This may be explained by organoleptic similarities, such as texture, color, and sweetness, which resembled the hypercaloric feed and made the cookies more appealing than the standard chow. Given the prior 12-week exposure to the hypercaloric diet, this similarity may have influenced food selection. This hedonic preference aligns with human sensory evaluations conducted during the product development phase, where both types of cookies demonstrated high acceptability (26). Nonetheless, changes in dietary distribution could also have contributed to inflammatory outcomes. Higher IL-6 levels were observed in the non-biotransformed cookie group compared to both the biotransformed cookie and cookie-free groups. Parallel differences were found in adipose tissue mass. These results suggest that cookie supplementation, when coupled with a dietary shift, may potentiate the bioactivity of *P. ostreatus* more effectively than in groups maintained on the hypercaloric diet without dietary transition. However, no significant differences emerged between the biotransformed and non-biotransformed subgroups. It is well established that mice on a hypercaloric diet may maintain or even gain weight despite similar intake volumes to those on standard diets. This is due to the high energy efficiency of fat-rich diets and an associated increase in lipid oxidation, which eventually promotes ectopic fat deposition (e.g., in liver or muscle) and the onset of insulin resistance (22).

Carbohydrate metabolism is typically disrupted under metabolic stress due to excessive glycemic load, persistent lipotoxicity, and insufficient intake of dietary fiber and bioactive compounds (26).

Although glucose levels during Phase 2 remained similar in STD and HYPSTD groups, glucose fluctuations were more pronounced in the HYPSTD group, particularly by week 20. These levels remain within the expected norm for mice fed hypercaloric diets, which typically present fasting glucose levels between 81 and 90 mg/dL (27). In this study, average glucose concentrations were 84.9 mg/dL across HYP groups and 98.6 mg/dL in the HYP CF group.

While no significant differences were observed in STD and HYP groups in glucose tolerance curves, minute 15 levels (glycemic peak) in the hypercaloric group reached 261 mg/dL, similar to values reported in studies using 8-16-week interventions. For example, in a study involving *Codonopsis pilosula* extract as a dietary intervention, mice exposed to a similar hypercaloric diet for 8 weeks exhibited peak glucose values of 261.2 mg/dL at minute 30 (28). In the present study, by minute 30, the biotransformed cookie group showed a more rapid return toward basal glucose values (198 mg/dL) compared to the CF group (223.6 mg/dL). These results reflect the established metabolic disturbances induced by hypercaloric feeding, insulin signaling impairment, reduced peripheral glucose uptake, and enhanced hepatic glucose production, often exacerbated by elevated triglyceride and free fatty acid levels (29).

These findings may also be partially explained by the physiological effects of the ingredients used in the intervention. Both beans and oats are rich in soluble dietary fiber, particularly resistant starches and *β*-glucans, which are known to slow gastric emptying and reduce the rate of intestinal glucose absorption, thereby attenuating postprandial glycemic excursions (30). Additionally, legumes provide bioactive compounds such as polyphenols and peptides that may enhance insulin sensitivity and modulate glucose transport through pathways involving glucose transporter type 4 (GLUT4) translocation (31).

Furthermore, the biotransformation process using *P. ostreatus* may enhance these effects by modifying the structural and chemical composition of the substrate. Fungal enzymatic activity can increase the availability of low-molecular-weight bioactive compounds, release bound phenolics, and partially degrade complex polysaccharides into more fermentable substrates. This may promote the production of short-chain fatty acids (SCFAs) through gut microbial fermentation, particularly propionate and butyrate, which are known to influence hepatic gluconeogenesis, improve insulin sensitivity, and regulate energy homeostasis (32). Collectively, these mechanisms could contribute to the improved glycemic recovery observed in the biotransformed cookie group.

Dyslipidemia is frequently associated with obesity, insulin resistance, and hepatic steatosis. High-energy diets increase *de novo* lipogenesis, promote visceral adiposity, and enhance hepatic triglyceride production and very-low-density lipoprotein (VLDL) secretion (33).

In our study, although Phase 1 exposure was insufficient to induce major lipid disturbances, subtle changes in lipid handling may have occurred, particularly under inflammatory conditions. For example, IL-6 has been shown to impair lipolysis and promote adipose tissue expansion (34), as observed in HYP groups that exhibited elevated IL-6 levels relative to HYPSTD and STD groups (35).

Metabolic dysfunction-associated steatotic liver disease (MASLD) is a common comorbidity of obesity, characterized by progressive lipid accumulation in hepatocytes and subsequent liver inflammation. Overfeeding, especially diets rich in simple sugars and fats, promotes hepatic de novo lipogenesis and macrophage infiltration, contributing to liver injury. However, a minimum of 40 weeks is generally required to elicit advanced hepatic changes in murine models (36). Thus, while macroscopic hepatic differences were noted, histopathological progression is likely still in early stages.

Regarding relative liver weight, in the hypercaloric groups, lower relative liver weights were observed with supplementation regardless of cookie type. Among these, the HYP group showed the smallest difference between the biotransformed and non-biotransformed subgroups, potentially due to prolonged exposure to the hypercaloric diet, an exposure absent in the other experimental groups, and a decreased preference for the biotransformed cookie during the final phase of the study, which led to increased consumption of the hypercaloric feed. The lower liver-to-body-weight ratios in the supplemented STD and HYP groups may reflect differences in hepatic lipid accumulation, overall energy intake, or other diet-related adaptations. However, liver weight alone cannot establish hepatic steatosis, inflammation, or tissue protection. Because histological examination and hepatic lipid quantification were not performed, these findings should be interpreted as changes in relative liver weight rather than evidence of reduced hepatic steatosis, inflammation, or liver injury.

Adipose tissue dysfunction is a key feature of diet-induced metabolic alterations. This dysfunction results primarily from chronic overfeeding, leading to disruption of lipid and carbohydrate metabolism and an increase in proinflammatory signaling. As adipose tissue stores expand, both structure and endocrine function are altered, contributing to insulin resistance and systemic inflammation (37).

A previous study in mice exposed to a high-fat diet for 8 weeks, followed by a low-fat diet for 7 weeks, showed that dietary reversal can reduce visceral fat mass; however, the inflammatory state induced by the initial diet may not be completely reversed. In the present study, the biotransformed group showed a numerical difference from the cookie-free group, but the comparison was not statistically significant. IL-6 levels were also lower in the standard biotransformed group than in the cookie-free group, supporting previous evidence of this cytokine’s role in inflammation (38). Increased adiposity may be associated with early inflammatory damage due to hypercaloric exposure. A study in C57BL/6 J mice on a high-fat diet for 52 weeks reported changes in IL-10 levels after week 24, attributed to increased expression of inflammatory genes (MCP-1, F4/80, IL-10) (36). This pattern was observed particularly in the HYPSTD group, especially when comparing the biotransformed-supplemented group to the unsupplemented one. This group is of special interest, as supplementation with the biotransformed cookie appeared to reduce adipose tissue mass more effectively in animals transitioning from a hypercaloric to a standard diet. This may indicate that cookies formulated with *P. ostreatus*-biotransformed flour can support lipid storage regulation in a favorable metabolic environment, especially when combined with dietary improvement. This pattern resembles the physiological adaptations typically observed during nutritional rehabilitation in humans, where dietary improvements and the intake of functional foods contribute to the restoration of homeostasis. In contrast, no comparable benefit was observed in the HYP group, likely due to the continued intake of a diet rich in lipids, which may have diminished the potential effects of supplementation. Notably, even in the STD group, composed of mice maintained under a healthy nutritional regimen, improvements in adipose tissue parameters were evident. This response may be attributable to the intrinsic bioactive properties of the cookie ingredients, including: oats (avenanthramides, *β*-glucans, ferulic and caffeic acids, flavonoids); black beans (anthocyanins, bioactive peptides, p-coumaric acid); and *P. ostreatus* mycelium (β-glucans, lectins, polyunsaturated fatty acids, pleurostrin) (39, 40).

Adipose tissue changes associated with hypercaloric diets have been previously described. In a study involving rats prone or resistant to obesity that were fed a high-fat diet for 7 weeks, subsequent analysis of inguinal and epididymal adipose tissue revealed hypertrophy and increased mass in both phenotypes. Additionally, elevated levels of cytokines such as IL-1*α*, IL-1β, IL-10, TNF-α, and IL-6 were documented (41). These findings support the notion that early metabolic and inflammatory changes occur even in genetically divergent responses to hypercaloric stress.

No significant differences were observed in the intestinal tissue recovered from the mice; however, a trend toward greater organ weight was noted in the groups supplemented with the biotransformed cookie. A study conducted on C57/BL6 mice subjected to small bowel resection and placed on a high-fat diet for 4 weeks showed a significant improvement in villus height and enterocyte proliferation after just 7 days of high-fat diet exposure. This increase was sustained throughout the 30-day period. In that study, food intake remained similar across groups, suggesting that the observed tissue changes were attributable to the administered diets, consistent with findings in the present study. These findings support the notion that fat may promote tissue repair and enterocyte proliferation, likely via the availability of nutrients in energy-dense diets rich in fiber or fat, possibly mediated by cluster of differentiation 36 (CD36) expression (42). This trend was also reflected in the average tissue weights in the groups fed the biotransformed cookie, which accounted for approximately 5% of the body weight in the STD, HYP, and HYPSTD groups. The observed effect may be associated with the enhanced nutrient availability provided by the cookies. While this effect was present in both B and NB cookie groups within the hypercaloric conditions, a more pronounced trend was observed in the HYPSTD B and STD B cookie groups.

The production of pro- and anti-inflammatory cytokines has been associated with the metabolic alterations typical of unhealthy diets, particularly when adipose tissue expansion leads to increased insulin resistance and macrophage infiltration. This effect is amplified when visceral fat accumulation occurs (43).

In the HYPSTD group, differences were observed between the NB group and the B and CF groups. The biotransformed group showed IL-6 concentrations similar to those in the cookie-free group, suggesting a possible reduction in this cytokine associated with the supplementation. Although a reduction in IL-6 was also seen in the HYP groups, the difference between NB and B groups was not significant. While the mechanism may differ, another study using *P. ostreatus* extract in macrophage and splenocyte cultures reported reduced IL-6 levels, likely via inhibition of nuclear factor kappa B (NF-κB) and activator protein 1 (AP-1) signaling (44). Similarly, previous studies have demonstrated that diets rich in saturated fats of animal origin can increase IL-6 levels within 10 weeks, with average concentrations reaching 17.6 pg./mL. These values are comparable to those recorded in our study for the HYP CF group (20.63 pg./mL) and HYPSTD CF group (9.93 pg./mL). The HYPSTD group, after switching to a standard diet, reached levels comparable to the STD CF group. IL-6 reductions were observed in the supplemented HYP groups, regardless of the biotransformation, compared to unsupplemented mice on a hypercaloric diet. The HYPSTD B group maintained high intake of the biotransformed cookies during Phase 2, especially starting at week 16. This might have influenced IL-6 regulation, especially in groups that underwent both dietary change and supplementation. This effect may be partially explained by the dietary shift, in which mice discontinued the hypercaloric diet, a factor known to contribute to reductions in IL-6 levels. The lower IL-6 concentrations observed in both supplemented HYP groups relative to HYP CF suggest that cookie supplementation was associated with modulation of this inflammatory marker under continued hypercaloric feeding. Because HYP B and HYP NB showed similar concentrations, this finding cannot be attributed specifically to biotransformation and may instead reflect components shared by both cookie formulations, differences in food intake, or other unmeasured factors. In the HYPSTD condition, IL-6 was lower in B than in NB mice; however, the similarity between B and CF indicates that the result may reflect the elevated value in the NB group rather than a general suppression of IL-6 by the biotransformed cookie. Components present in oats, beans, and P. ostreatus, including *β*-glucans and phenolic compounds, may influence inflammatory signaling. Consistent with this possibility, extracts of *Pleurotus* species, including *P. ostreatus*, have demonstrated inhibitory activity against NF-κB/AP-1 signaling in cellular models (45).

Regarding IL-10, no marked differences were observed between the STD and HYP groups, but a reduction was noted in the HYPSTD group. This could be attributed to the dietary change in that group, as *P. ostreatus* supplementation in the other groups did not affect IL-10. Within the HYPSTD group, differences emerged between supplemented and non-supplemented subgroups. These findings suggest a temporal pattern in consumption behavior, with increased cookie intake observed in the HYPSTD B and HYPSTD NB groups by week 12, accompanied by a reduced proportion of standard diet consumption. This trend diminished by week 16, followed by a renewed increase toward week 20. The outcomes in IL-10 concentrations may be influenced by ingredients in the cookies (e.g., butter, egg), which could trigger inflammatory responses when consumed in higher quantities. A reduction in IL-10 has been associated with inflammation and obesity, primarily in adipose tissue. IL-10 can suppress IL-6 and TNF-*α* levels, both of which were elevated in the HYPSTD NB and B groups, respectively. This suggests a possible compensatory regulation. Additionally, IL-10 may play a paradoxical role in adipose tissue, possibly promoting insulin resistance and thermogenesis (46). In the STD and HYP groups, IL-6 and IL-10 remained elevated, potentially reflecting mutual regulation. In contrast, the HYPSTD group showed variability in IL-6 levels, mirroring fluctuations in IL-10 (47). A study in genetically predisposed rats found decreased IL-10 levels, suggesting more efficient repair in models resistant to inflammation (41). Persistent inflammation may result in adipose tissue hyperplasia and macrophage infiltration, with M1 macrophages driving pro-inflammatory cytokine production, and M2 macrophages driving anti-inflammatory responses. The predominance of M1 macrophages in obesity promotes chronic inflammation (48). In this study, combined dietary change and supplementation led to IL-6 regulation, especially when comparing NB and non-cookie subgroups. The inverse association between IL-6 and IL-10 may reflect cytokine regulation, although its direction and mechanism cannot be determined from these data. This effect seems more evident in the HYPSTD group, where diet change without concurrent supplementation may have driven this response pattern, in contrast to the STD and HYP groups.

TNF-*α* levels showed no significant differences between supplemented and non-supplemented groups. However, slight changes were seen in the B and CF subgroups within the HYPSTD group. These results are consistent with IL-6 patterns in the same group. IL-10 may have compensated for reduced IL-6. In inflammatory states, IL-6, IL-10, and TNF-α may all increase, with IL-6 exhibiting the greatest fluctuations during systemic inflammation (49). This may reflect chronic inflammatory regulation. A study using obese mice and human adipose cells reported that high TNF-α levels may promote IL-6 production, possibly explaining the cytokine trends observed (50).

Notably, the HYPSTD group underwent unique dietary shift, transitioning from a hypercaloric diet to a standard diet. This may have gradually attenuated the effects of prior metabolic disruption. One limitation of this study is the relatively short duration of hypercaloric exposure (<26 weeks), which may have led to subtler outcomes compared to longer interventions. Nonetheless, the observed tissue and cytokine changes still suggest selected dietary, tissue, and inflammatory responses associated with the hypercaloric intervention. In the HYPSTD groups, cytokine regulation appears to have been modified by the new dietary regimen, potentially influencing both positive and negative inflammatory responses.

This study has several limitations that should be considered when interpreting the findings. First, the relatively small sample size per group (*n* = 5) may have limited statistical power to detect subtle metabolic differences across the nine experimental groups. Second, only male mice were included; therefore, sex-specific responses cannot be excluded. Third, baseline biochemical measurements were not obtained at week 0 because the animals entered the protocol at approximately 3 weeks of age, a stage at which invasive blood sampling requires special caution due to limited circulating blood volume and welfare considerations. Fourth, the duration of hypercaloric exposure may not have been sufficient to induce more severe metabolic dysfunction in all parameters. Finally, histological analyses and mechanistic approaches such as microbiota profiling or metabolomic characterization were not included. Future studies should address these limitations using larger cohorts, both sexes, longer interventions, and deeper molecular phenotyping.

## 5 Conclusion

In this preclinical study, 12 weeks of hypercaloric feeding altered energy and macronutrient intake but did not produce significant differences in body weight, fasting glucose, triglycerides, or overall glucose tolerance compared with the standard diet. During the subsequent supplementation phase, the observed responses varied according to dietary condition and outcome. In mice maintained on the standard diet, consumption of the biotransformed cookie was associated with lower liver- and adipose-tissue-to-body-weight ratios compared with the cookie-free control. Under continued hypercaloric feeding, both cookie-supplemented groups exhibited lower relative liver weight and serum IL-6 concentrations than the cookie-free group. Because these effects were observed with both the biotransformed and non-biotransformed formulations, they cannot be attributed exclusively to the biotransformation process.

In mice transitioned from the hypercaloric to the standard diet, the biotransformed-cookie and cookie-free groups exhibited lower IL-6 concentrations than the non-biotransformed-cookie group. However, the metabolic, tissue, and cytokine findings were not consistent across all outcomes, and TNF-*α* and IL-10 concentrations were largely comparable among treatments. Collectively, these results indicate that supplementation was associated with selected tissue and inflammatory outcomes rather than a generalized improvement in metabolic status. They also demonstrate that the response to the cookie formulations depended on the underlying dietary condition.

These findings provide preliminary *in vivo* evidence supporting further investigation of *Pleurotus ostreatus*-biotransformed bean flour as a potential functional ingredient for bakery products. Nevertheless, the small group size, reduced sample availability for cytokine analyses, inclusion of only male mice, limited development of the intended metabolic phenotype, and absence of histological and mechanistic analyses restrict the conclusions that can be drawn. Future studies should use larger cohorts of both sexes and a longer, validated hypercaloric intervention to determine whether the observed responses are reproducible in a more clearly established metabolic phenotype. Standardizing the administered cookie dose would also help distinguish treatment effects from differences in voluntary food consumption. Histological evaluation of the liver and adipose tissue, measurement of hepatic lipid accumulation and adipocyte morphology, and analysis of tissue inflammatory-cell infiltration are needed to clarify the biological significance of the relative tissue-weight differences. In addition, microbiota profiling, short-chain fatty-acid analysis, metabolomic characterization, and targeted quantification of bioactive compounds would help identify mechanisms associated with P. ostreatus biotransformation. Evaluation of inflammatory and metabolic signaling pathways, including NF-κB, AP-1, and GLUT4-related responses, would further test the mechanisms proposed in this study. Finally, product-standardization, dose–response, safety, sensory, and shelf-life studies should precede controlled human interventions.

## Data Availability

The raw data supporting the conclusions of this article will be made available by the authors, without undue reservation.

## Supplementary material

SUPPLEMENTARY FIGURE S1 Metabolic parameters during Phase 1 of the experimental design. **(A)** Oral glucose tolerance test (OGTT) curves measured at week 4 in mice fed standard (STD) or hypercaloric (HYP) diets. Blood glucose concentrations were evaluated at 0, 15, 30, 45, 60, and 120 minutes following oral glucose administration. **(B)** Fasting glucose levels during weeks 4 and 12. Data are presented as mean ± SEM. No statistically significant differences were observed between dietary groups during Phase 1 (*p* > 0.05).

SUPPLEMENTARY FIGURE S2 Serum triglyceride concentrations during Phase 1 of the experimental design in mice fed standard (STD) or hypercaloric (HYP) diets. Measurements were performed at weeks 4 and 12. Data are presented as mean ± SEM. No statistically significant differences were observed between groups (*p* > 0.05).

SUPPLEMENTARY FIGURE S3 Summary heatmap integrating metabolic status, organ ratios, and inflammatory biomarkers across the nine experimental groups. Data represent standardized group means (z-scores). Warmer colors indicate higher relative values, and cooler colors indicate lower relative values for each variable. This visualization facilitates global comparison of intervention effects among groups.
